# Herbivory and misidentification of target habitat constrain region-wide restoration success of spekboom (*Portulacaria afra*) in South African subtropical succulent thicket

**DOI:** 10.7717/peerj.11944

**Published:** 2021-08-11

**Authors:** Marius L. van der Vyver, Anthony J. Mills, Mark Difford, Richard M. Cowling

**Affiliations:** 1 Botany Department, Nelson Mandela University, Port Elizabeth, Eastern Cape, South Africa; 2 Soil Science, University of Stellenbosch, Matieland, Western Cape, South Africa

**Keywords:** Ecological restoration, Herbivory, *Portulacaria afra*, Spekboom, Subtropical thicket, Target habitat, Restoration planting, Biome-scale

## Abstract

Restoration of degraded subtropical succulent thicket, via the planting of *Portulacaria afra* (spekboom) truncheons, is the focus of a public works programme funded by the South African government. The goals of the programme, which started in 2004, are to create jobs, sequester carbon, restore biodiversity, reduce erosion, improve soil water holding capacity and catalyse private sector investment for upscaling of restoration. Here we report on a region-wide experiment to identify factors that can improve project success. Measures of success were survivorship and annual aboveground biomass carbon sequestration (ABCsr) of spekboom truncheons some 33–57 months after planting—starting in March 2008—into 173 fenced plots (0.25 ha) located throughout the global extent of spekboom thicket vegetation. We also collected data for 18 explanatory variables under the control of managers, and an additional 39 variables reflecting soil physical and chemical characteristics and rainfall patterns post restoration, all beyond the influence of managers. Since the latter covariates were available for only 83 plots, we analysed the two data sets separately. We used a prediction rule ensemble to determine the most important predictors of restoration success. There was great variation in percentage survivorship (median = 24, range = 0–100%) and ABCsr (median = 0.009, range = 0–0.38 t C ha^−1^ yr^−1^). The model using management variables explained less variance (53%) in survivorship than the model incorporating additional soil and rainfall covariates (62%). ABCsr models were better fits (78 and 88% variance explained, respectively). All model configurations identified browse intensity as a highly influential predictor of restoration success. Predicted success was highest for plots located in target habitat; however, only 45% were thus located, suggesting the need for expert input and habitat modelling for improving target habitat identification. Frost exposure was another important predictor influencing all models but was likely a consequence of locating sites off target habitat. Sites planted on equatorward slopes during the warm season showed reduced carbon sequestration, possibly due to elevated soil moisture stress associated with high radiation loads. Physiographic factors associated with improved restoration success were location on sloping ground (reduced frost exposure), increasing longitude (more warm-season rainfall) and increasing latitude (less frost coastwards). Few trends were evident among post-restoration climatic factors beyond the control of managers. Higher rainfall during the year post restoration had a negative impact on carbon sequestration while higher rain during the early months post restoration had a positive effect on both carbon sequestration and survivorship. Soil factors showed little importance for the survivorship model, whereas silt content, % K and Mg CEC emerged as predictors of carbon sequestration. Our results have direct relevance for improving the success of landscape-scale restoration projects envisioned for the ca. 8,930 km^2^ of degraded spekboom thicket.

## Introduction

**Figure 1:**
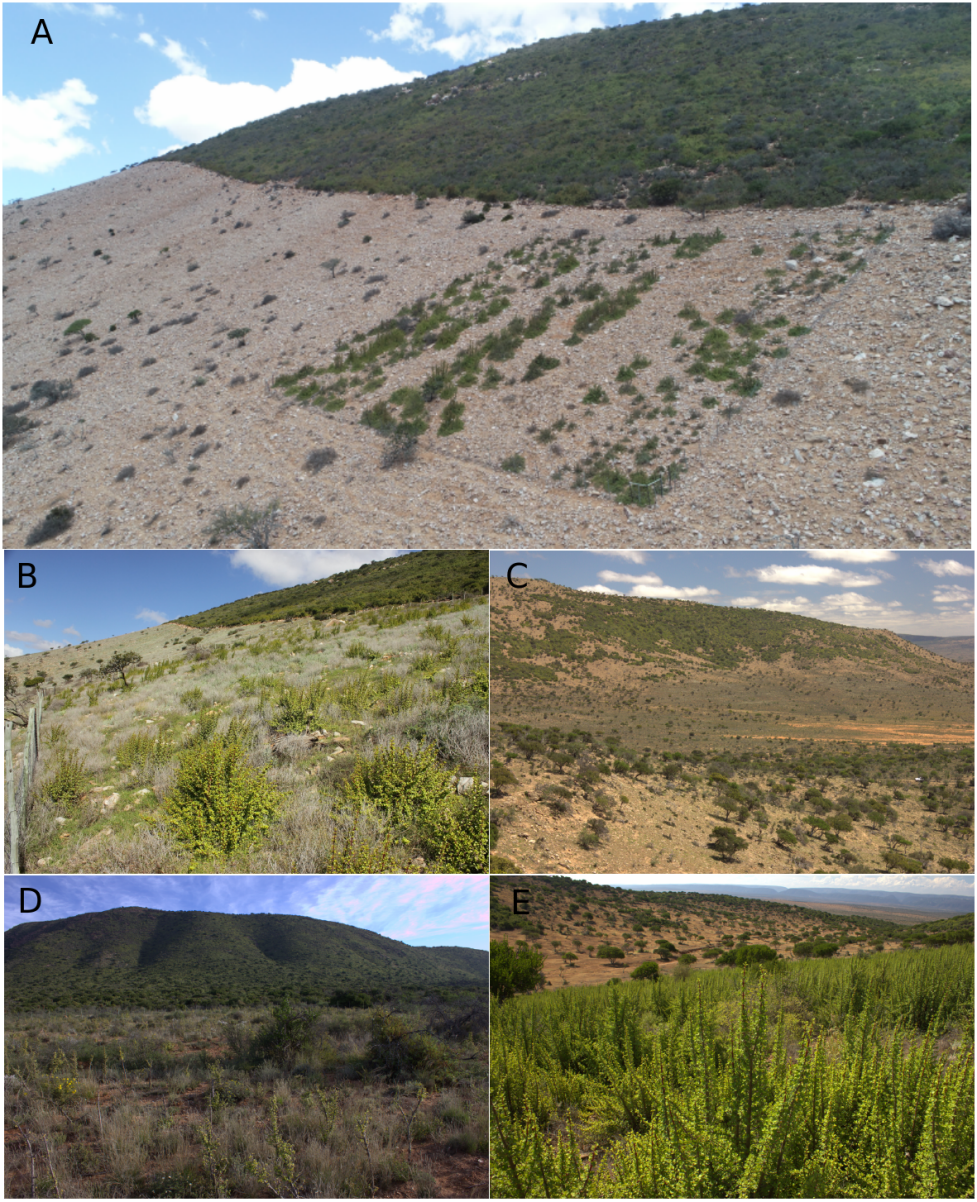
Different cover states of spekboom thicket (intact, degraded, restored) as well as adjacent, karroid vegetation. (A) A thicket-wide plot (TWP) planted on the degraded side of a fenceline contrast with intact spekboom thicket upslope. (B) A view from within a TWP showing planted spekboom on degraded target habitat with a fenceline contrast with intact spekboom thicket in the background. (C) Patchy intact spekboom thicket under heavy browsing pressure on the hilltop bordering Nama-Karoo vegetation on the valley floor with degraded spekboom thicket in the foreground showing remnant trees (mainly *Pappea capensis*) forming a pseudosavanna. (D) A TWP planted in Nama-Karoo vegetation on frost-prone bottomlands with spekboom thicket on frost-free slopes in the background. (E) A TWP planted in degraded spekboom thicket (*i.e.,* target habitat), which also covers the slope in the background. Photos: M. van der Vyver.

Degraded subtropical thicket of southern South Africa is the focus of a state-funded public works programme, the Subtropical Thicket Restoration Programme (STRP) (Mills et al., 2010). This region-wide restoration project aims to restore degraded habitat *via* the planting of *Portulacaria afra* (hereafter spekboom) truncheons to create jobs, sequester carbon, restore biodiversity, reduce erosion, improve soil water holding capacity and catalyse private sector investment to upscale restoration (Fig. 1) (Mills & Cowling, 2006; Mills & Cowling, 2010; Van Luijk et al., 2013; Polak & Snowball, 2019). Spekboom is a succulent shrub or low tree (2–5 m) that is very drought resistant, establishes readily from cuttings, and is capable of sequestering impressive amounts of carbon for a semi-desert plant (Mills & Cowling, 2006; Van der Vyver et al., 2013; Mills & Cowling, 2014; Guralnick & Gladsky, 2017), much of which is contributed to the soil pool *via* leaf litter (Lechmere-Oertel et al., 2008; Mills & Cowling, 2010). Spekboom dominates in drier forms (<550 mm yr^−1^) of Subtropical Thicket in frost-free areas (Vlok, Euston-Brown & Cowling, 2003). Hereafter, we term this spekboom thicket.

Protocols for, and endpoints of thicket restoration using spekboom are based on research from just two sites, both located in the centre of the spekboom thicket biome. Research conducted at these sites suggests that thicket vegetation structure, biodiversity and aboveground carbon stocks can be restored in about 30–50 years (Mills & Cowling, 2006; Van der Vyver et al., 2013). Moreover, planting established saplings of woody canopy species (*e.g.*, *Pappea capensis, Searsia longispina*) of spekboom thicket as part of the restoration protocol is cost-ineffective, owing to almost total mortality of these species, compared with almost zero mortality of transplanted, nursery-grown spekboom truncheons (Van der Vyver et al., 2012).

Results from two sites near to one another are insufficient for identifying efficient and effective restoration protocols for a biome-wide programme. Moving from the scale of site to landscape in restoration initiatives is difficult (Brudvig, 2011; Rodrigues et al., 2011; Menz, Dixon & Hobbs, 2013; Aavik & Helm, 2018; Molin et al., 2018). Insights gleaned from restoration outcomes at a local site where many factors are strictly controlled, may not hold over the wider range of the target habitat. This may be due to differences in soils, climate and other physiographic factors, or variation in restoration implementation by the many managers and teams involved in landscape-level projects (Rodrigues et al., 2009; Halme et al., 2013; Wortley, Hero & Howes, 2013; Crouzeilles et al., 2016). Herbivory, and associated impacts such as trampling by domestic and indigenous animals, which is usually excluded from site-bound restoration experiments, may be a crucial determinant of success in large-scale implementation where the costs of excluding herbivores may be prohibitive (Sweeney, Czapka & Yerkes, 2002; Daws & Koch, 2015; Thyroff, Burney & Jacobs, 2019).

Misidentification of target habitat is another factor that may confound restoration success at the biome scale, especially if planners and implementers lack the requisite ecological expertise to identify the target habitat. Implementing in non-target habitat can waste much effort but, more importantly, can compromise biodiversity and ecosystem services as is the case in misidentifying intact grassland or savanna as degraded forest requiring restoration (Veldman et al., 2015; Bond, 2016).

The identification of suitable target habitat is particularly difficult in degraded thicket where livestock-induced degradation has blurred boundaries between Subtropical Thicket and two interdigitating karroid biomes, namely Succulent Karoo and Nama-Karoo (Hoffman & Cowling, 1990; Vlok, Euston-Brown & Cowling, 2003; Sigwela et al., 2009). Since degraded thicket superficially resembles karroid shrubland (Fig. 1) (Lechmere-Oertel, Kerley & Cowling, 2005), identifying the boundaries between these biomes in degraded landscapes requires knowledge of the flora, particularly the karroid shrub and ephemeral (mainly annuals) components (Vlok, Euston-Brown & Cowling, 2003).

**Figure 2:**
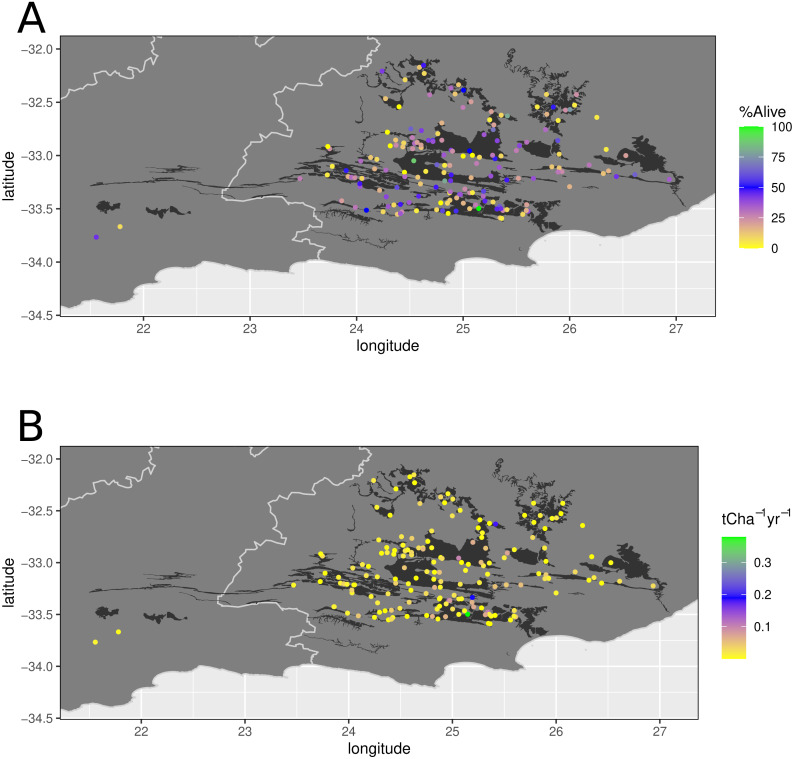
Location of 173 thicket wide plots in relation to spekboom thicket vegetation types. Location of 173 thicket wide plots in relation to spekboom thicket vegetation types according to Vlok, Euston-Brown & Cowling (2003) (black background). The top panel represents the survivorship (percentage alive) and bottom panel the aboveground carbon sequestration rate per annum (t C ha^−1^ yr^−1^).

To improve restoration protocols for degraded thicket at the landscape scale, the STRP decided in 2007 to implement a region-wide restoration experiment. Three hundred 0.25-ha sites were located throughout the global distribution of spekboom thicket, spanning a rainfall seasonality gradient from east (more warm-season rainfall) to the west (more cool-season rainfall), and from the more equitable coastal regions in the south, to the more continental climates of the north (Vlok, Euston-Brown & Cowling, 2003) (Fig. 2). Implementation of the experiment was undertaken by a government agency tasked with sourcing and training contracted teams of unemployed local labourers. The STRP acted as a planning and advisory body, and consisted of contracted scientists and academics, representatives from implementing agents, land managers and government policy makers. STRP scientists designed the layout of the experimental plots, which consisted of 13 different treatments planted as replicated rows of spekboom truncheons within each plot. To ensure that plots were in degraded thicket and not karroid vegetation, scientists provided training on identifying these states to the three regional (east, central, west) managers who were tasked—amongst other duties—with negotiating with landowners on the placement of experimental plots.

In this study we report on an assessment of the experiment three to five years (33–57 months) after the establishment of the plots, starting in March 2008. Our interest was in determining the factors affecting restoration success or effectiveness as measured in terms of percentage survivorship of planted truncheons and aboveground biomass carbon sequestration rate per annum (ABCsr). We were particularly interested in the relative role of factors related to human actions (*e.g.* location of plot in target habitat, protection of plots from herbivory, influence of the restoration contractor) and those related to factors beyond the control of managers, namely rainfall patterns after planting, and the role of soil physical and chemical factors. We conclude by providing recommendations to improve the effectiveness of restoration protocols.

### Spekboom thicket distribution and ecology

The study area comprises the global distribution of spekboom thicket, delimited by the Fish River drainage in the east and the Gouritz River drainage in the west (Fig. 2) (Vlok, Euston-Brown & Cowling, 2003). This region is highly heterogeneous in terms of topography, climate, geology and soils, and is home to eight biomes which interdigitate in complex ways (*e.g.*, Vlok, Euston-Brown & Cowling, 2003; Cowling & Potts, 2015). Spekboom thicket is often, but not exclusively associated with relatively deep, shale-derived and mostly rocky soils on sloping ground (Vlok, Euston-Brown & Cowling, 2003). Annual rainfall is bimodally distributed, with peaks in spring and autumn. In the west, more rain falls in the cooler months while the east may experience appreciable summer rain. Mean annual precipitation ranges from 200 mm in the western, inland regions to 550 mm in the eastern, coastal regions (Vlok, Euston-Brown & Cowling, 2003). Localised flash floods and prolonged droughts are not uncommon. Summers are very hot with temperatures exceeding 40 °C not uncommon; winters are mild, but the nights may be cold, and frost occurs regularly in bottomlands as a result of cold-air pooling (Duker et al., 2015a; Duker et al., 2015b). The geology is dominated by rocks associated with the Cape Supergroup (quartzitic sandstones, shales), Karoo Supergroup (mudstones, shales) and Uitenhage Group (mudstones, conglomerates) (Vlok, Euston-Brown & Cowling, 2003). Other than the Cape sandstones, which yield infertile sands, the other geologies yield mainly moderately fertile and relatively clay-rich soils (Campbell, 1983; Cowling & Potts, 2015; Cawthra et al., 2019).

Spekboom thicket comprises a dense, 2–5 m-tall, tangle of hedge-forming shrubs; low, multi-stemmed trees; dwarf, shrub and tree succulents; lianas and vines; and geophytes (Vlok, Euston-Brown & Cowling, 2003) (Fig. 1). Spekboom is usually the canopy dominant but coexists with a high diversity of non-succulent trees and shrubs, notably *Euclea undulata, Pappea capensis, Schotia afra* and *Searsia* spp. Thicket communities and landscapes are rich in species and include many local and regional endemics (Cowling, 1983; Johnson, Cowling & Phillipson, 1999). Spekboom is a large (2–5 m), hedge-forming shrub, with both succulent stems and leaves. It can rapidly shift from predominantly C3 to CAM photosynthetic pathways (Guralnick & Gladsky, 2017) in response to increased water stress, longer photoperiods (Guralnick, Rorabaugh & Hanscom, 1984a), and increased daytime temperatures (Guralnick, Rorabaugh & Hanscom, 1984b). This rapid switch allows it to make use of sporadic rainfall events, even during a prolonged drought (Guralnick & Ting, 1987).

Intact spekboom thicket ecosystems store carbon in excess of 200 t C ha^−^^1^ (measured up to a soil depth of 50 cm), a remarkable feature for a xeric ecosystem (Mills et al., 2005a; Van der Vyver & Cowling, 2019). Most of this carbon stock is associated with spekboom (Mills & Cowling, 2006; Lechmere-Oertel et al., 2008); its dense canopy provides the relatively cool and dry conditions necessary for the accumulation of the high levels of soil carbon (Lechmere-Oertel, Cowling & Kerley, 2005; Lechmere-Oertel, Kerley & Cowling, 2005; Mills & Cowling, 2010) and maintenance of biodiversity (Wilman et al., 2014). Comparisons of degraded and intact sites reveal carbon losses of more than 80 t C ha^−1 ^ (Mills et al., 2005a; Mills et al., 2005b). These losses are evident from the decrease in above-ground biomass, but also manifest in a massive reduction in soil organic carbon content (Mills & Fey, 2004; Mills & Cowling, 2010).

Heavy, continuous browsing by goats throughout the spekboom thicket region has resulted in the widespread mortality of the highly palatable spekboom as well as the loss of many other species, notably dwarf succulent shrubs; the net result is the transformation of the dense thicket community to an open savanna-like vegetation (Hoffman & Cowling, 1990; Kerley, Knight & De Kock, 1995) (Fig. 1). The transformed state comprises scattered and stressed individuals of trees (mainly *E. undulata* and *P. capensis*) in a matrix of ephemeral herbs and short-lived shrubs and grasses (known locally as “opslag”), whose abundance tracks rainfall events (Hoffman & Cowling, 1990; Lechmere-Oertel, Cowling & Kerley, 2005; Thompson et al., 2009). Approximately 75% of spekboom thicket (spekboom thicket and spekboomveld of Vlok, Euston-Brown & Cowling (2003) (8,931 km^2^ out of a total of 11,950 km^2^) had been altered in this manner by the the turn of the century. The emergence of voluntary carbon-markets, carbon taxation and related climate mitigation mechanisms has created opportunities for recovering restoration costs and providing additional economic incentives for landowners to restore degraded land under their keep (Alexander et al., 2011; Feng et al., 2013; Ma et al., 2014). Our research is aimed at providing evidence-based recommendations for improving the effectiveness of spekboom thicket restoration.

## Materials & Methods

### Experimental plots

Three regional project managers (denoted P, S and Y) were given training by ecological experts to identify restoration sites within the region delimited by the extent of spekboom thicket according to Vlok, Euston-Brown & Cowling (2003). The training included identification of species indicative of degraded thicket and karroid vegetation. Where doubt existed on the original vegetation of a site, managers were advised to make use of existing fence-line contrasts between intact and degraded sites to establish experimental plots (Fig. 1). In the absence of these, managers were instructed to place sites on a similar position in the landscape to the nearest stand of intact thicket (which needed to be sufficiently close to enable non-mechanised harvesting and distribution of truncheons for restoration), and always to remain within spekboom thicket habitat as delimited by Vlok, Euston-Brown & Cowling (2003).

Three hundred plots were established between March 2008 and October 2009 by teams contracted and trained by the national Department of Environmental Affairs’ Working for Woodlands programme, *via* its implementing agent, the Gamtoos Irrigation Board in agreement with participating landowners. Managers and contracted teams were given training to enable them to harvest spekboom cuttings of suitable dimensions from nearby intact stands, and plant these in multiple rows according to 13 different treatments within a fenced herbivore exclosure of 0.25 ha (50×50 m). Fencing comprised a 1.2 m high wire-mesh stabilized by steel droppers and wooden posts at the corners. Two strands of wire were added to increase the height to circa 1.4 m. Each treatment was replicated by 2–4 rows within each plot, and distinguished according to various truncheon stem diameters, planting depths and the application of extraneous treatments, namely the addition of water during planting, sealing of top-cut surfaces, applying root hormone, and combinations of these. Treatment rows were assigned randomly. The randomised design created problems for data collection as many treatments in many plots could not be identified. Here we focus on only one of the treatments: truncheons of ±22.5 mm in stem diameter, planted 2 m apart at a depth of 15 cm, with no extraneous treatments applied. Three rows of 27 individuals allocated to this treatment were randomly located within each plot. We chose this treatment because the wider truncheon spacing was unique to it, thereby enabling its easy identification in the field. Accordingly, this treatment provided the largest sample size of all treatments, which suited the aims of this study, focused as they are on region-wide patterns. Furthermore, this treatment represents the planting protocol recommended by the STRP for the restoration of degraded spekboom thicket at the time when the experiment was implemented.

### Data collection

The response variables for this experiment were survivorship (%Alive) and aboveground biomass carbon sequestration rate (ABCsr) per year of planted truncheons. With verbal permisison from landowners, data were collected by a scientific services company (Conservation Support Services) between June 2012 and January 2013, some 33–57 months after planting. Over the same period, one of the authors (M.L. van der Vyver) visited the same plots to collect a range of metadata on site (Supplementary Material Fig. S1). Out of the 300 plots planted, only 173 had sufficient information to be of use in this study. As we describe below, two subsets of these plots were used in different analyses, owing to unavailability of the full set of explanatory variables for all these 173 plots. Survivorship of planted truncheons was quantified by counting all alive, dead and missing truncheons within each planted row in each plot. Height, canopy diameter and stem diameter of nine planted truncheons, spaced in clusters of three at the beginning, middle and end of each row, were recorded using measuring sticks and digital callipers. Where less than nine truncheons survived in a row, only measurements from those that were alive were recorded, regardless of position within the row. In total, we assessed 1,403 truncheons with the full range of available explanatory variables and 3,114 with only explanatory variables directly under the control of a project manager.

We estimated carbon sequestration rate (ABCsr) using the best fitted allometric model of Van der Vyver & Cowling (2019) based on spekboom height and canopy dimensions (Eq. (1)). Thus:

(1) \begin{eqnarray*}AB{C}_{P.afra}=\mathrm{exp}(-11.15)~\cdot (\mathrm{\pi }C{r}^{2}~\cdot ~Hgt)^{0.58}~\cdot ~D{W}_{ratio}~\cdot ~{C}_{frac}\end{eqnarray*}

Equation (1) estimates aboveground biomass carbon (ABC) (kg) from the canopy radius (Cr), measured as half the mean of two canopy diameter measurements (cm), and the total height (Hgt) (cm) of the plant. The result was multiplied by the dry:wet ratio (DW_*ratio*_) estimated for spekboom at 0.271 (see Van der Vyver & Cowling, 2019), and by a general standard estimated fraction of carbon from dry woody biomass (C_*frac*_) at 0.5 (Thomas & Malczewski, 2007). We derived an ABCsr estimate from the truncheon measurements for canopy diameter and height per row (max *n* = 9), by taking the mean ABC of all measured truncheons per planted row multiplied by the number of surviving truncheons counted in that row. ABCsr per plot was computed by dividing mean ABCsr per row by the age of that restoration plot, measured from the date of planting up to the date of sampling (months), and extrapolating the result to tonnes of carbon per hectare per year (t C ha^−^^1^ yr^−^^1^).

We compiled data for 57 variables likely to explain survival and growth responses of spekboom truncheons. The acronyms for corresponding explanatory variables are provided in the text and a full list is given in Table S1. We placed special emphasis on modelling restoration effectiveness in terms of covariates that can be controlled by managers involved in landscape-level projects located throughout the spekboom thicket region. However, we were also interested in the explanatory power of the edaphic and rainfall covariates, which are not under management control. Thus, we ran separate models for the two data subsets: (1) topographic and management variables (173 plots and 535 rows), and (2) topographic, management, soil and rainfall variables (83 plots and 254 rows).

Topographic covariates included aspect, recorded with a compass in the middle of a plot; slope, estimated within 5°  on a 10-point scale and ranging from 0°–45°; landform (landf), estimated on a numerical four-point scale of increasing altitude, from bottomlands (1), footslope (2), midslope (3) to crest (4); elevation (ele) in masl; longitude (lon), which in this region is representative of a west-east gradient of increasing warm-season rainfall; latitude (lat), indicative of a gradient of declining moisture and increasing temperature extremes moving inland from the coast (the three last-mentioned were read from a handheld GPS-device).

Spekboom thicket is restricted to frost-free environments and spekboom is particularly vulnerable to frost damage (Duker et al., 2015a). Therefore, the severity of frost damage on planted truncheons is likely to be a reliable predictor of the occurrence of the target habitat for restoration, and hence the success of managers to identify this. We reported evidence of frost damage (frostb) as a binary Y—N variable for each plot, as inferred from the condition of surviving truncheons. A shriveled stem, often with spontaneous flaking, sometimes lichen-infested, and blackened leaf discoloration, were used as indicators of frost (Duker et al., 2015b) (see Fig. S1 for examples of frost damage).

Spekboom is a relatively palatable shrub, readily browsed by domestic and wild herbivores (Stuart-Hill, 1992; Sigwela et al., 2009). Therefore, it was important to model the effects of browsing intensity on restoration success. Since each landowner agreed to maintain their plot enclosures in a good condition throughout the duration of the experiment, we regarded browsing impacts (browse) as a management issue. We estimated browse impacts on a numerical four-point scale (1 = none, 2 = light, 3 = medium, 4 = heavy), based on the intensity of browsing damage observed on live truncheons at each plot (see Fig. S2 for examples of browse intensity). Browsing damage on planted truncheons is easily distinguishable from damage due to frost (Duker et al., 2015b). Signs of wildlife presence (wlife) inside a plot, evidenced by direct encounters during sampling, tracks, scat and damaged and askew fences; and signs of domestic stock entry (DomE), similarly evidenced by direct encounter, scat, tracks and mohair residues inside the plot, were recorded as binary Y—N predictors. We also coded as a binary variable whether there was a hole in the exclosure fence large enough for a small browser to enter (holefence), and whether the entrance gate to the plot was left open (gateO). To assess overall condition of the exclosure fence (fenceC), we allocated to each plot a score ranging from 0 where no part of the original fence was left standing to 5, where the fence retained original intended integrity or better. A score of 3 or below signified either a breach or hole in the fence large enough, or a fence-pole sufficiently askew, to allow access to the plot by a medium-sized herbivore (common duiker *Sylvicapra grimmia* or domestic goat *Capra aegrargus hircus*).

We assessed whether the plots were in the correct (spekboom thicket) habitat in two ways. First, we determined whether the plot was located within the appropriate habitat according to the 1:100 000 scale vegetation map of Vlok, Euston-Brown & Cowling (2003) (STEPvt) produced for the Subtropical Thicket Ecosystem Project. Plot vegetation was categorised as spekboom thicket, vegetation types lacking spekboom (exclusively karroid types), or mosaics of spekboom thicket with these other vegetation types. Second, we categorised the target vegetation in the field (habitat), based on extant vegetation patterns in the near vicinity, position in the landscape, and the presence of indicator plant species. Good indicators of target habitat are the remnant tree succulent *Aloe speciosa* and a dense cover after good rains of weedy ephemerals such as *Mesembryanthemum guerichianum* and *Atriplex lindleyi* ssp. *inflata* (alien invasive) (Vlok, Euston-Brown & Cowling, 2003). On the other hand, the trees *Vachellia karoo* and *Aloe ferox*, and shrubs such as *Becium burchellianum, Pentzia incana* and *Pteronia incana* are indicative of non-target karroid vegetation.

Additional restoration management factors considered as categorical variables were the identity of the responsible managers (P in the central study domain, S in the eastern domain and Y in the west), and the season (spring, summer, autumn, winter) in which the plot was planted (seasonP).

We used soil data from four topsoil (0–20 cm) samples collected from the centre of the plot by the planting team. The samples were analysed by an accredited laboratory (BemLab Pty Ltd, Stellenbosch, South Africa). Seventeen soil variables were analysed using standard laboratory protocols; the corresponding method is provided in parentheses after listing the variable together with the acronym, where appropriate. These variables comprised pH (potassium chloride), stone volume percentage (stone) (water displacement), available P (P_mg) (Bray II, but if pH > 7.0 then Olsen method), extractable K (K_mg) (Colwell), soil cation exchange capacity (cmolc kg^−1^) and base saturation of four bases, Na, K, Ca and Mg (the former depicted as follows: [base]_mg and the latter as [base]_pc), % organic carbon (C_pc) (Walkley-Black method), % total N (N_pc) (Kjeldahl) and the % clay, silt and sand content determined by mechanical methods.

Rainfall data were obtained for 40 climate stations in the study domain from the South African National Weather Service. Local landowners provided data for an additional two stations. A climate station was deemed representative of a plot if it occurred in a 30-km radius of that plot. Given the relatively steep rainfall gradients of the region, this distance is not ideal. However, the mean altitudinal difference between the weather stations and their associated plots was only -45 m, while the mean vertical and horizontal distances were 111 m and 15 km, respectively. The rainfall variables assessed included total precipitation (mm) during the month in which planting took place (mm1mnt), but also over a period of 3, 6, 12 and 18 months since planting took place (mm3—6—12—18mnt). We did not quantify rainfall in the period prior to planting although we agree that this variable may have had significant explanatory power. Seasonal rainfall variables included the total rainfall recorded, after 6, 12 and 18 months since planting, during the summer (Dec–Feb) (summ6—12—18), spring months (Sep–Nov) (spr6—12—18), winter (Jun–Aug) (wnt6—12—18) and autumn (Mar–May) (atm6—12—18) since planting. Since none of the experimental plots experienced measurable rainfall during the six summer months immediately after planting, this variable was discarded. We also included total rainfall over the same time intervals since planting as the other seasonal precipitation variables, recorded during the warmer (Sep–Feb) (sumr6—12—18) and cooler months (Mar–Aug) (wnt6—12—18).

### Statistical analysis

We used RuleFit3™, a prediction rule ensemble (Friedman & Popescu, 2003; Friedman & Popescu, 2008) to determine the most important predictors of restoration efficacy, and fit sparse models in terms of simple prediction rules (coefficients) as decision trees. The method enables high prediction accuracy and excellent interpretability by identifying simple rule sets as coefficients ordered in terms of importance to a fitted linear model (Friedman & Popescu, 2003; Friedman & Popescu, 2008).

We ran models for the two data subsets described above (topographic and management, and all explanatory variables)as paired models for the two response variables (%Alive and ABCsr). For each of the two models, we applied three different penalties (ridge, elastic net and lasso) to account for collinearity and other biases. We compared models in terms of goodness-of-fit statistics, variable importance ratings (Friedman & Popescu, 2008) and rule sets (decision trees). All RuleFit3™  models were implemented without any additional observation weights and with an average of four terminal nodes in generated trees. A full 10-fold, cross-validated error for each model was also obtained. All statistical analyses were performed with R (R Core Team, 2019), and the packages RuleFit3™  for R (Friedman & Popescu, 2012), pre (Fokkema, 2020) and akima (Akima & Gebhardt, 2016) while most graphics were compiled with the package ggplot2 (Kahle & Wickham, 2013).

## Results

### Patterns of restoration efficacy in relation to target habitat and herbivory

According to the 1:100 000-scale STEP map, 65% of the 173 plots were planted in spekboom thicket and its mosaics, while 35% were planted in non-spekboom thicket habitat (Fig. 2). In contrast, only 45% of plots were in target habitat (spekboom thicket and its ecotones) as assessed by us in the field.

The mean truncheon survivorship across all the planted rows (*n* = 711) in all the plots assessed (*n* = 173) was 28% (median = 24, range 0–100%), while mean aboveground biomass carbon sequestration rate (ABCsr) amounted to 0.02 t C ha^−1^ yr^−^^1^ (median = 0.009) (Fig. 2). As with survivorship, variation in ABCsr was high, ranging from zero to 0.379 t C ha^−^^1^ yr^−^^1^.

**Figure 3:**
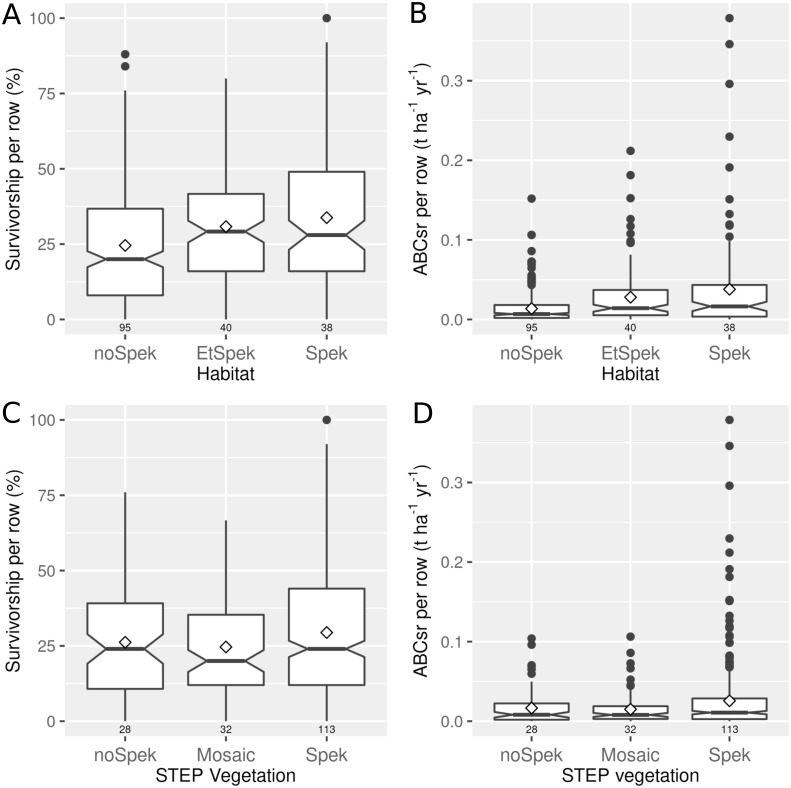
Percentage survivorship and aboveground biomass carbon sequestration rate (ABCsr) of planted spekboom (*Portulacaria afra*) truncheons in 173 experimental (thicket wide) plots. Boxplots showing the percentage survivorship (left panels) and aboveground biomass carbon sequestration rate (ABCsr) (right panels) of planted spekboom (*Portulacaria afra*) truncheons in 173 experimental (thicket wide) plots. The top panels show results according to field-scale delineation of spekboom habitat. Spek refers to spekboom thicket habitats, EtSpek to ecotones or transition zones between spekboom thicket and other habitats, and noSpek refers to non-spekboom thicket habitats. Bottom panels show the results for the regional-scale (1:100 000) STEP delineation of target habitat (Vlok, Euston-Brown & Cowling, 2003). Sample size in terms of number of planted rows and plots are indicated above and below boxes, respectively. Mean values are indicated by diamonds in the boxes.

Plots in field-identified spekboom thicket and its associated ecotones recorded mean truncheon survival of 34% (median = 28%) and 31% (median = 29%), respectively (Fig. 3 top left panel). Lower survival (mean = 25%; median = 20%) was recorded in plots located in non-target habitat. A similar pattern emerged for ABCsr, with the highest values recorded in target habitat (mean = 0.038 t C ha^−^^1^ yr^−^^1^, median = 0.02) and its ecotones with adjacent vegetation (mean = 0.028 t C ha^−^^1^ yr^−^^1^, median = 0.014), and lowest in non-target habitat (mean = 0.014 t C ha^−^^1^ yr^−^^1^, median = 0.007) (Fig. 3 top right panel). When plot vegetation was characterised according to the STEP map delineation, restoration success did not differ markedly among vegetation characterisations (Fig. 3 bottom panels)]. Both survivorship and carbon sequestration were lowest for mosaic vegetation, and of similar magnitude for spekboom thicket non-thicket vegetation.

**Figure 4:**
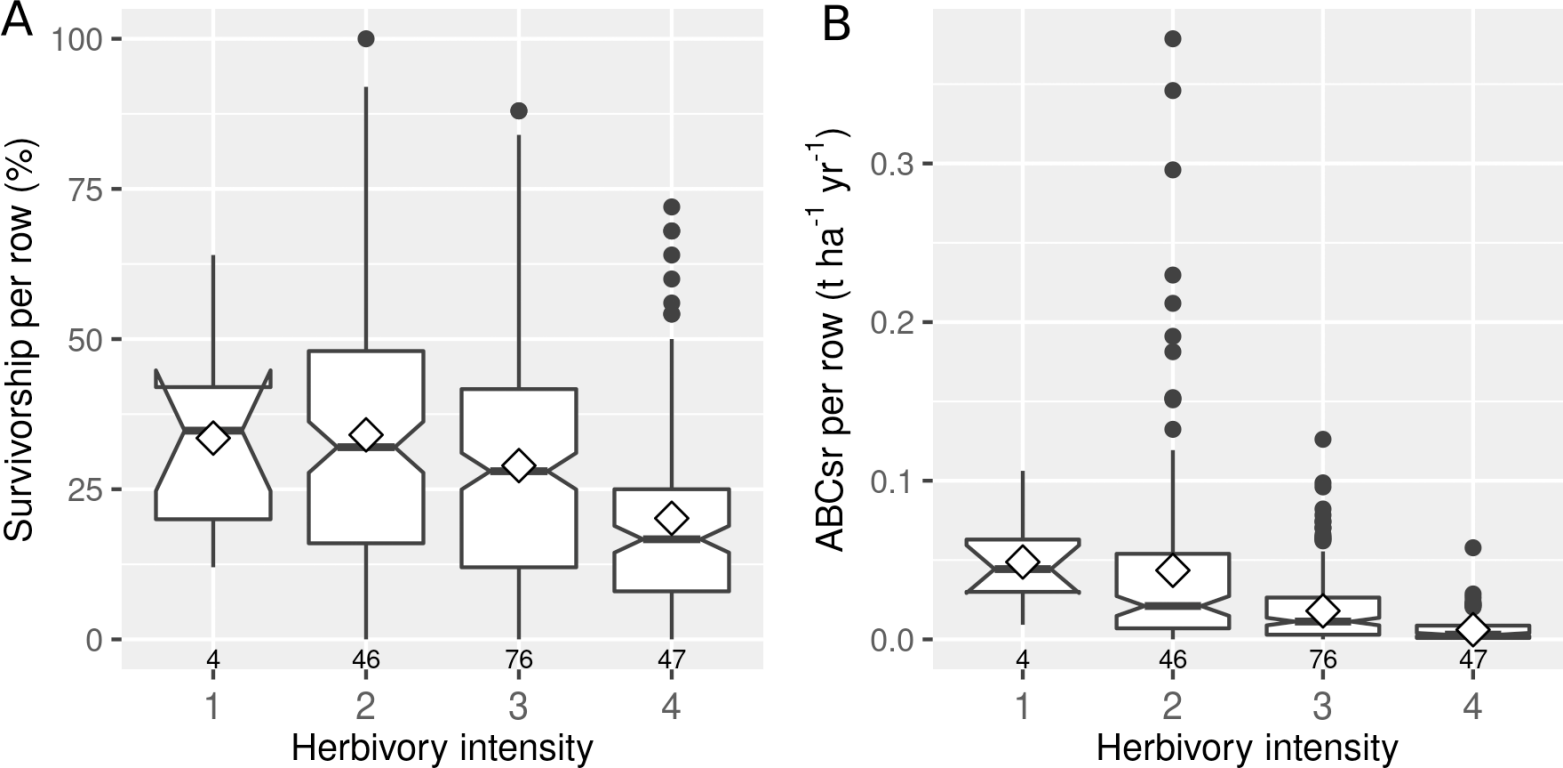
Percentage survivorship and aboveground biomass carbon sequestration rate (ABCsr) per row of planted spekboom (*Portulacaria afra*) truncheons in 173 experimental plots. Boxplots showing percentage survivorship (left panel) and aboveground biomass carbon sequestration rate (ABCsr) (right panel) per row of planted spekboom (*Portulacaria afra)* truncheons in 173 experimental plots. Browsing intensity scores as follows: 1 = none; 2 = light; 3 = medium and 4 = heavy. Sample size in terms of number of planted rows and plots are indicated above and below boxes, respectively. Mean values are indicated by diamonds in the boxes.

The intensity of herbivory had a negative effect on both the survivorship (%Alive) and carbon sequestration (ABCsr) of spekboom truncheons (Fig. 4). Mean survivorship increased from 20% (median = 17%) under conditions of heavy browse exposure to 34% (median = 35%) under very light browsing. Corresponding values for mean ABCsr were 0.006 (median = 0.003 t C ha^−^^1^ yr^−^^1^) and 0.049 t C ha^−^^1^ yr^−^^1 ^(median = 0.044 t C ha^−^^1^ yr^−^^1^).

### Model statistics

Table 1 shows the model statistics for the models fitted to the two different subsets of predictor variables, defined as follows: topographic and management variables (*n* = 173 plots); and with soil and rainfall variables added (*n* = 83 plots). These were further subject to three levels of penalty, as defined in Table 1. When fitted to topographic and management covariates, the survivorship (%Alive) models showed an overall weaker fit (50–67% variance explained) than the carbon sequestration (ABCsr) models (28–88%). Apart from the carbon sequestration models penalized with ridge regression, where the more variable-laden model could only explain 28% of the variance, the addition of the soil and rainfall covariates to the models improved the fit of both the survivorship and carbon sequestration models by a similar degree.

**Table 1:** Statistics of RuleFit3^TM^ models fitted with three regularization penalties to two subsets of the data. Statistics of RuleFit3TM models fitted with three regularization penalties to two subsets of the data, namely topographical and management variables (*n* = 173 plots), and these variables plus edaphic and rainfall variables (*n* = 83 plots).

model^a^	pen^b^	response^c^	nplot^d^	nvar^e^	nrow^f^	mae^g^	rmse^h^	rmse.n^i^	sd.n^j^	%varExp^k^	cv.mse^l^	cv.mse.se^m^	cv.mae^n^	cv.mae.se^o^
rfA_n173_a0	0.0	pcAlive	173	18	535	11.28	14.00	0.69	0.62	0.52	3.00e+02	1.85e+01	1.39e+01	4.47e−01
rfA_n173_a07	0.7	pcAlive	173	18	535	10.87	13.86	0.68	0.64	0.53	2.79e+02	1.73e+01	1.34e+01	4.32e−01
rfA_n173_a1	1.0	pcAlive	173	18	535	11.43	14.34	0.71	0.61	0.5	2.78e+02	1.71e+01	1.33e+01	4.36e−01
rfA_n83_a0	0.0	pcAlive	83	57	254	8.99	11.47	0.57	0.70	0.67	3.03e+02	3.15e+01	1.34e+01	6.96e−01
rfA_n83_a07	0.7	pcAlive	83	57	254	9.81	12.37	0.62	0.67	0.62	2.40e+02	2.31e+01	1.21e+01	6.07e−01
rfA_n83_a1	1.0	pcAlive	83	57	254	8.96	11.42	0.57	0.73	0.67	2.66e+02	2.73e+01	1.28e+01	6.38e−01
rfC_n173_a0	0.0	ABCsrt	173	18	535	0.01	0.02	0.61	0.66	0.63	7.07e−04	1.23e−04	1.61e−02	9.14e−04
rfC_n173_a07	0.7	ABCsrt	173	18	535	0.01	0.02	0.46	0.84	0.78	7.98e−04	1.32e−04	1.67e−02	9.86e−04
rfC_n173_a1	1.0	ABCsrt	173	18	535	0.01	0.02	0.46	0.84	0.79	5.98e−04	1.13e−04	1.54e−02	8.21e−04
rfC_n83_a0	0.0	ABCsrt	83	57	254	0.02	0.04	0.85	0.24	0.28	2.11e−03	7.12e−04	2.53e−02	2.41e−03
rfC_n83_a07	0.7	ABCsrt	83	57	254	0.01	0.02	0.35	0.88	0.88	1.06e−03	3.26e−04	1.88e−02	1.66e−03
rfC_n83_a1	1.0	ABCsrt	83	57	254	0.01	0.02	0.35	0.87	0.88	6.66e−04	1.36e−04	1.69e−02	1.22e−03

**Notes.**

a RuleFit model tested.

b penalty - 0 = ridge, 0.7 = elastic net, 1 = lasso.

c response variable. %Alive = survivorship percentage, ABC sr = aboveground biomass sequestration rate (t C ha^−1^ yr^−1^).

d number of plots.

e number of predictors.

f number of planted rows.

g mean absolute error.

h root mean square error.

i normalised root mean square error, using the standard deviation of the response.

j normalized standard deviation.

k % variance explained = 1-rmse.n2.

l 10-fold cross-validated mean square error.

m standard error of 10-fold cross-validated mean square error.

n 10-fold cross-validated mean absolute error.

o standard error of 10-fold cross-validated mean absolute error.

Since the three penalization models identified similar suites of important explanatory variables (unpublished results) and generated similar model statistics, we chose, as a compromise between parsimony and complexity biases (Zou & Hastie, 2005), to display the outputs of the model with elastic net penalization (Table 1). Explained variances for these models were as follows: survivorship: topographic and management variables (53%), survivorship: all variables (62%); carbon sequestration: topographic and management variables (78%), carbon sequestration: all variables (88%).

### Models using topographic and management covariates

**Figure 5:**
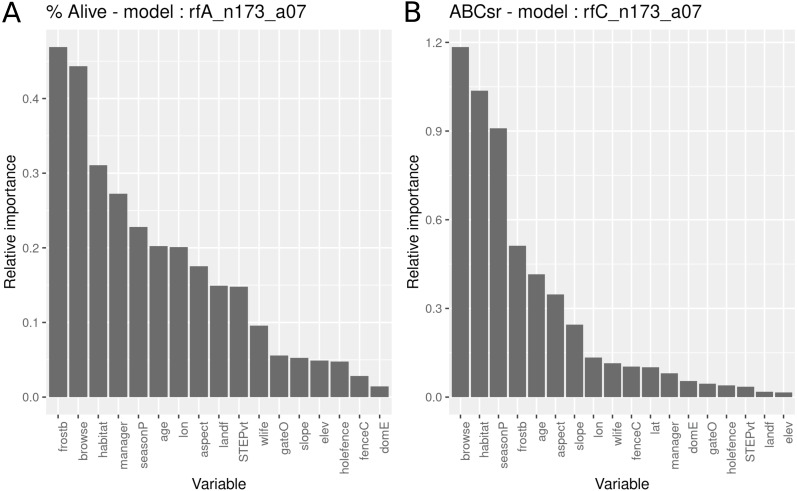
Relative importance of variables for two RuleFit3^TM^ models fitted to topographic and management variables (*n* = 173 plots, *n* = 535 rows, *p* = 18) for predicting survivorship and carbon sequestration rate. Relative importance of variables for two RuleFit3^TM^ models (rfA_n173_a07 and rfC_n173_a07) (see Table 1) fitted to (A) topographic and management variables (*n* = 173 plots, *n* = 535 rows, *p* = 18 covariates) for predicting survivorship and (B) carbon sequestration rate (ABCsr). Both models are regularized with elastic net (*α* = 0.7). Model statistics are shown in Table 1. Explanations of acronyms are provided in Table S1 as well as in the text.

The survivorship model identified frost exposure and browse intensity as the two most important predictors, followed by habitat (field-scale identification of vegetation), restoration manager, season of planting, restoration age, longitude, aspect and landform (Fig. 5). The carbon sequestration model (ABCsr) identified browse intensity and habitat as the two most important variables. Next in importance were season of planting and frost exposure, followed by restoration age, and aspect. Both models selected all six variables related to browsing impacts (browse, wilife, domE, gateO, holefence and fenceC).

**Figure 6:**
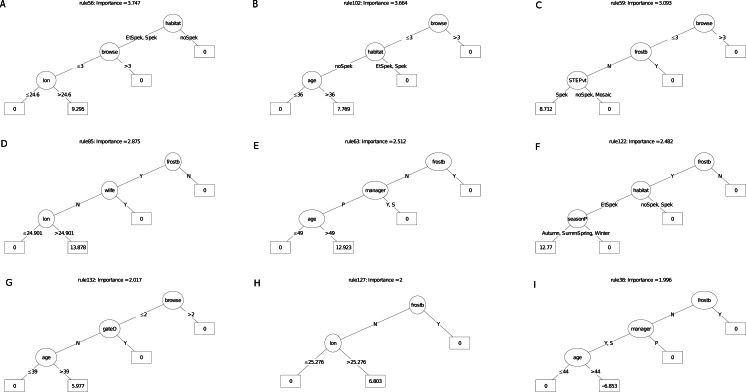
(A–I) An ensemble of the nine most important decision trees for the survivorship RuleFit3^TM^ model (rfA_n173_a07) built on topographic and management factors, with age as a covariate. An ensemble of the nine most important decision trees for the survivorship RuleFit^TM^ model (rfA_n173_a07) (Table 1) built on topographic and management factors, with age as a covariate. Rule coefficients are shown in the bottom rule of each tree. Explanations of acronyms are provided in Table S1 and the text.

**Figure 7:**
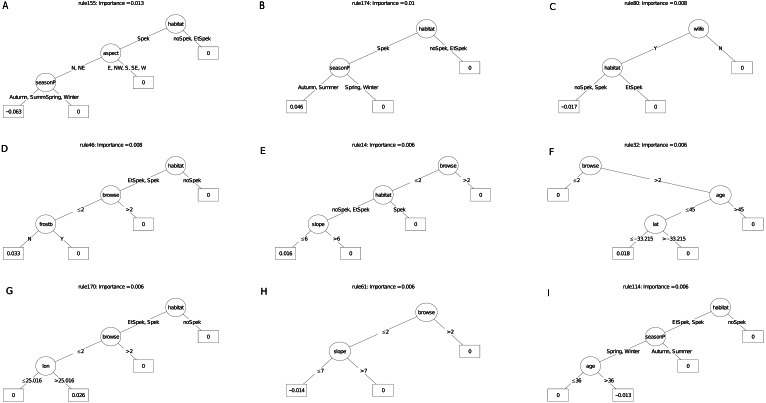
(A–I) An ensemble of the nine most important decision trees for the carbon sequestration RuleFit3^TM^ mode. An ensemble of the nine most important decision trees for the carbon sequestration RuleFit3^TM^ model (rfC_n173_a07) (Table 1) built on topographic and management factors, with age as a covariate. Rule coefficients are shown in the bottom rule of each tree. Explanations of acronyms are provided in Table S1 and the text.

Figures 6 and 7 illustrate an ensemble of the nine most important decision trees ranked on their rule coefficients, for the two RuleFit models (Fig. 5). These decision trees are useful for capturing the direction of relationships between explanatory and response variables and enable assessment of the interactions amongst variables based on rules arising from the linear models. For example, the most important decision tree for the survivorship model (Fig. 6), identified plots located on target habitat (spekboom thicket and adjacent ecotones) that experience moderate or less browse intensity and east of 24.6°  longitude, as the most important multiplier of survival (coefficient = 9.3%). Overall, the most frequent rules in the decision trees associated with better survival were reduced exposure to browsing (including evidence of wildlife presence) (five trees), frost exposure and location in target habitat (including the STEP classification) (three decision trees each). Post restoration age was identified in four models where, except for one, a time-since-planting of >36 month was identified as having a positive effect on survivorship. Plot location east of ca. 24.6°  (more warm-season rainfall) identified with increased survival (three decision trees). The identity of manager appeared in two decision trees. One of these identify one manager with increased survival in older plots (coefficient = 13%), another tree associated negative survival with the other two managers; note that managers were geographically divided according to longitude where the best performing manager occupied the central position. Planting in autumn and summer were identified as multipliers of survivorship in association with frost-prone ecotones within target habitat (one decision tree).

For the carbon sequestration (ABCsr) model (Fig. 7), the most important predictive rule set associated decreased sequestration (coefficient = −0.07 t C ha^−1^ yr^−1^) in plots located field-identified target habitat (excluding ecotones), on equator-facing slopes and planted in the warmer seasons. As was the case for the survivorship decision trees, location in target habitat (four decision trees), reduced browsing intensity, including the presence of wildlife (four decision trees), lower latitudes (≤ −33.2), longitude (east of ca. 25°) and frost exposure (one decision tree) emerged as the most frequent positive multipliers of ABCsr. Season of planting was identified in three rule sets; results, however, were contradictory. One rule sets showed a negative effect on carbon sequestration of truncheons planted in the warmer summer and autumn months while the other two showed a positive effect. Post restoration age was positively associated with increased carbon sequestration in one tree and negatively associated in another. One decision tree identified lower browse intensity with slopes less than 35° steep with negative carbon sequestration, another selected slopes less than 30° with elevated ABCsr.

### Models using all covariates

**Figure 8:**
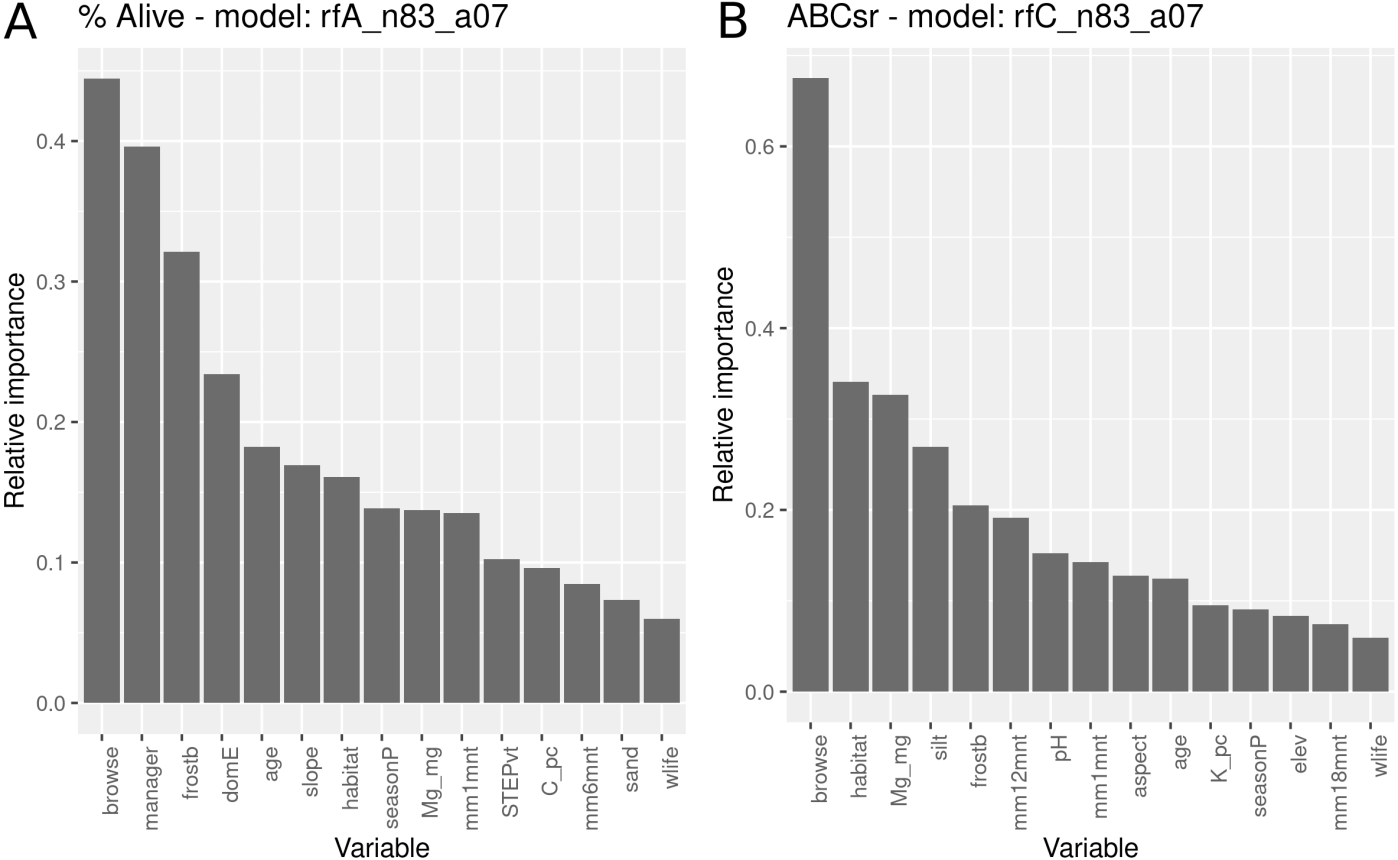
Relative importance of variables for two RuleFit3^TM^ models fitted to topographic, management, soil and rainfall variables for predicting survivorship and carbon sequestration rate. Relative importance of variables for two RuleFit3^TM^ models (rfA_n83_a07 and rfC_n83_a07) (see Table 1) fitted to (A) topographic, management, soil and rainfall variables (*n* = 83 plots, *n* = 254 rows, *p* = 57 covariates) for predicting survivorship and (B) carbon sequestration rate (ABCsr). Both models are regularized with elastic net (*α* = 0.7). Model statistics are shown in Table 1. Explanations of acronyms are provided in Table S1 as well as in the text.

The survivorship model using all covariates identified browse intensity as the most important explanatory variable (Fig. 8), followed by the manager, exposure to frost, domestic animal access, post restoration age, slope angle and field-assessed target habitat. Evidence for entry of domestic animals (typically goats) and wildlife were two additional variables selected in the model that reflect indirect exposure to browsing. Unlike the models with only topographic and management variables (Fig. 5), location in target habitat did not emerge as a highly important covariate. Soil variables were identified as having relatively low importance for predicting survivorship.

**Figure 9:**
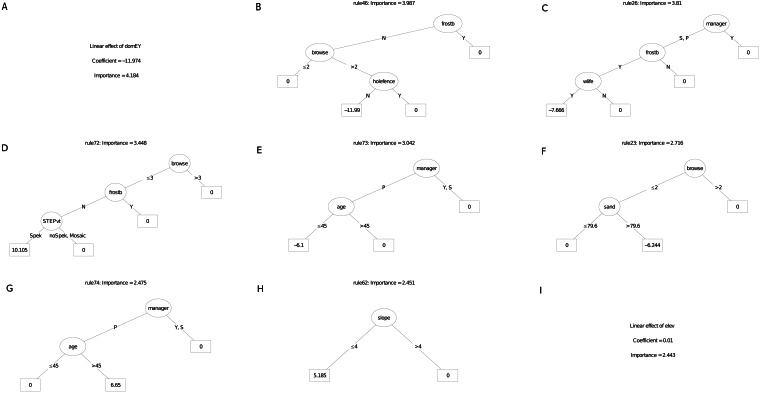
(A–I) An ensemble of the nine most important decision trees for the survivorship RuleFit^TM^ model (rfA_n83_a07) built on all explanatory variables, with age as a covariate. An ensemble of the nine most important decision trees for the survivorship RuleFit^TM^ model (rfA_n83_a07) (Table 1) built on all explanatory variables, with age as a covariate. Rule coefficients are shown in the bottom rule of each tree. Explanations of acronyms are provided in Table S1 and the text.

Of the nine most important decision trees for the survivorship model, the best rule set (coefficient = 12.2%) selected plots planted under manager P that were older than 45 months. (Fig. 9). The restoration manager appeared in four decision trees but without clear trends, as the second most important decision tree identify managers S and P with negative survival. Exposure to frost appeared in two trees, both as a negative multiplier of survival. The positive effect of reduced browsing, and the negative effect of entry by domestic animals, were each reflected in different trees. Two trees identified a positive linear effect of target habitat with increased survival, one according to our field classification of ecotones in target habitat, the other according to the STEP classification. Another tree identified the negative effect of lower rainfall (ca. 31.6 mm in the month of planting) on survivorship; another identified slope angled 25° and less with increased survivorship. The other variable to emerge was post restoration age (ca. 45 - month threshold), which had a positive effect on survivorship.

The carbon sequestration (ABCsr) model identified browse intensity as the most important covariate (Fig. 8). Target habitat (field-identified), soil Mg cation exchange capacity (CEC), silt percentage, frost exposure and total rainfall within a year post planting followed, with importance values ranging between 30% and 50% lower than browse intensity (Fig. 8). Other variables identified by the model were soil pH, the amount of rainfall recorded in month of plot establishment, and aspect.

**Figure 10:**
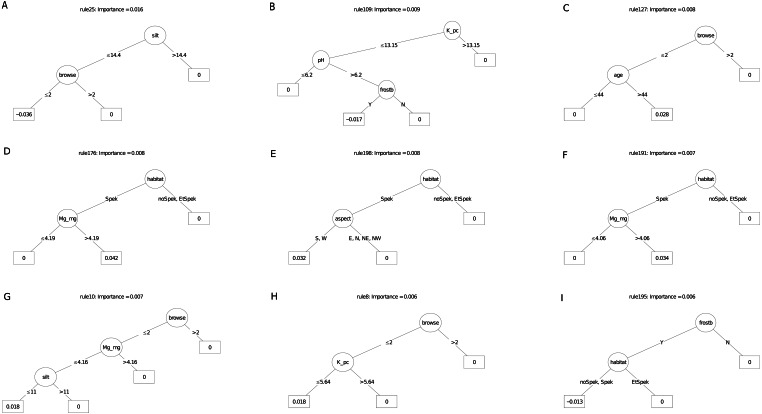
(A–I) An ensemble of the nine most important decision trees for the carbon sequestration RuleFit3^TM^ model built on tall explanatory variables, with age as a covariate. An ensemble of the nine most important decision trees for the carbon sequestration RuleFit^TM^ model (rfC_n83_a07) (Table 1) built on tall explanatory variables, with age as a covariate. Rule coefficients are shown in the bottom rule of each tree. Explanations of acronyms are provided in Table S1 and the text.

The best rule set (rule 198) amongst the nine most important decision trees arising from the carbon sequestration model, identified lower soil silt percentage (14.4% threshold) in low browsing conditions, as a negative multiplier of carbon sequestration (Fig. 10). Less than 244.5 mm of rain in the first year after planting had a surprisingly positive effect. Lower browsing intensity had an overwhelmingly positive influence on sequestration (five trees), while the presence of wildlife combined with frost exposure negatively affected sequestration rate in another tree. Older restored plots were associated with increased carbon sequestration in two trees. Sites receiving rainfall in the first year of planting of less than ca. 240 mm recorded positive multipliers of carbon sequestration in two trees (Fig. 10); In contrast, rainfall of 16 mm or more recorded within the month of planting was a positive multiplier of carbon sequestration. One ruleset identified plot location in field-identified spekboom habitat as a positive multiplier of carbon sequestration. Given the high importance of soil variables in the parent model (Fig. 8), it is not surprising that these appeared as nodes in four of the nine selected decision trees. Mg (cation exchange capacity) above a threshold of ca 3.22–4.19 cmolc  kg^−^^1^ was a positive multiplier of carbon sequestration in two trees. Positive effects were also found amongst the rule sets for lower levels of soil K.

## Discussion

Achieving success in region-wide restoration projects is challenging since protocols are invariably developed based on the outcomes of experiments conducted at a local as opposed to a biome scale. At the local scale, the array of controlling factors is likely to be only a subset of the full spectrum and the effects of many explanatory variables can be controlled or manipulated (Miller & Hobbs, 2007; Menz, Dixon & Hobbs, 2013). Such control or manipulation is often not feasible at larger spatial scales. By adopting a region-wide approach and implementing and evaluating an experiment replicated across the full extent of environmental variability in spekboom thicket vegetation, we have derived some insights that are likely to benefit future spekboom restoration projects. Our principal conclusions are that restoration sites must be protected from browsing, especially by wildlife, and that every effort must be made to ensure that they are located in the appropriate spekboom thicket habitat. However, we acknowledge that our results were contradictory in places (*e.g.*, browsing both promoted and suppressed carbon sequestration, depending on the context) and our interpretations and recommendation must be treated with caution.

Our results show that mean survivorship of planted truncheons per plot is relatively low (28–34%), regardless of topographic site selection, management, and soil and rainfall variables. The relatively low variance explained (50–53%) by the survivorship models when only topographic, management and post-restoration age factors were modelled, suggest that survivorship is not well predicted with this subset of variables. When soil and rainfall variables were added, the survivorship model fits were slightly better (62–67% variance explained). The relatively good fit (63–79%) for the aboveground carbon sequestration (ABCsr) models that incorporated topographic and management variables, showed similar improvement (except for models penalised with ridge regression) with the addition of soil and rainfall predictors (28–88% variance explained). This suggests that topographic and management related factors, all within the control of the restoration practitioner, are important determinants of rates of carbon sequestration. In the sections below, we explore how our findings can be used by managers to improve the success of landscape-level restoration initiative in spekboom thicket.

### Herbivory

All models identified high browse intensity as an influential predictor of restoration success, although based on the frequency of its identification in the nodes of rule sets, this factor appeared to play a more important role in carbon sequestration than for truncheon survival. Surprisingly, one decision tree associated with the model fitted to all explanatory variables showed lower intensity browsing as having a negative effect on sequestration. The fact that all of the variables related to a specific aspect of herbivore management (i.e., domestic stock entry, signs of wildlife, fence condition, and open gates) emerged amongst the most important predictors in the fitted models based on only topographic and management variables, suggests that none of these variables can in isolation predict the intensity of browse that was observed. Few landowners maintained their exclosure fences during the experiment, despite agreeing to do so. Our data suggest that browsing by both wildlife—most likely Greater kudu (*Tragelaphus strepsiceros*)—and livestock, had the greatest impact on restoration success. This inference is supported by the variable “evidence of wildlife” being the most important predictor of carbon sequestration among browsing-related covariates where only topographic and management factors were considered. The fencing for the exclosures was insufficiently high to prevent access by Greater kudu, which require fences >2.4 m to constrain their movements. Entry of domestic animals into plots emerged as the fourth most important variable for predicting survivorship in models where all variables were considered.

The fact that herbivory by wildlife—especially Greater kudu—is prevalent over most of the thicket biome today poses a challenge for effective spekboom thicket restoration. Since the mid-1960s there has been a large increase in wildlife populations in both commercial rangelands and protected areas, coupled with a massive boom in game ranching since the 1990s (Sims-Castley et al., 2005; Carruthers, 2008). While intact spekboom thicket can support a relatively high density of wildlife, including megaherbivores such as African elephant *Loxodonta africana* and black rhinoceros *Diceros bicornis* (Kerley, Knight & De Kock, 1995; Mills & Cowling, 2014), newly planted truncheons—especially in degraded thicket where spekboom is absent—provide a novel source of nutrition for browsers such as Greater kudu. As is the case for many other restoration contexts (Stanton-Clements, Koch & Daws, 2013; Parsons et al., 2007; Sweeney, Czapka & Yerkes, 2002), newly planted truncheons in plots of a few hectares in size should be protected from indigenous browsers until they are resilient to this impact. Doing this will require the erection of specialised fencing and will be costly. Large-scale planting of truncheons over thousands of hectares could greatly diminish the effects of Greater kudu on individual truncheons owing to browse satiation. This issue requires further research. The entry of domestic stock into plots where fences were compromised by wildlife (typically Greater kudu breachings) and not subsequently maintained is common. In some cases, experimental plots provided emergency feed for stock farmers battling degradation and drought conditions, and domestic animals were intentionally brought in.

### Target habitat

Our results showed that the correct identification of target habitat is of critical importance for restoring degraded spekboom thicket. Although 65% of plots were planted within the regional-scale (1:100,000) STEP delineation of spekboom thicket and its mosaics, only 45% fell within spekboom thicket target habitat (including ecotonal vegetation) as identified in the field by the authors. The scale of the STEP map was too coarse to be an effective guide for managers to identify suitable sites (see also Cumming, Cumming & Redman, 2006; De Knegt et al., 2010; Guerrero et al., 2013), with this covariate having minimal influence on restoration success.

A further constraint on the identification of target habitat by managers is the difficulty of distinguishing in the field, even for professionals, between degraded spekboom thicket and heavily grazed karroid vegetation. The training provided to regional managers stressed the importance of landform in guiding site choice, notably that frost-prone bottomlands should be excluded unless there was evidence of spekboom thicket at the same position in the landscape. Indeed, our results showed that restoration success was low in bottomland plots (see also Duker et al., 2020). On the positive side, planting outside of target habitat is unlikely to produce a biome-switch—as is the case when savanna or grassland is misidentified as forest habitat (Veldman et al., 2015; Bond, 2016)—since truncheons are likely to succumb to frost, and without adequate protection, to herbivory.

Our field-scale habitat categorization was highly effective as a predictor of restoration success, being the second most important predictor for the carbon sequestration (ABCsr) models, and always influential in the truncheon survival models. This highlights the need for using regional ecological experts to delineate target restoration habitat in the absence of a sufficiently fine-scaled map (Thompson et al., 2009). It also highlights the potential for applying predictive habitat distribution modelling to this problem (De Knegt et al., 2010).

### Other important variables under management control

Given the bimodality of rainfall in the spekboom thicket region, with relatively reliable spring and autumn rainfall peaks (Vlok, Euston-Brown & Cowling, 2003), we expected that planting in these seasons would enhance survivorship and carbon sequestration. However, we found no clear pattern regarding the effects of season of planting on carbon sequestration. Contrary to expectations, planting in the warmer seasons (summer and autumn) when soil water deficits are likely to be greatest, had a positive influence on survivorship. We did find consistent evidence that planting on equator-facing slopes in the warmer months reduced carbon sequestration rates, probably because these slopes are subject to the highest radiation loads, and hence soil moisture stress, during the austral summer and transitional seasons (Schulze, 1975). However, other than this outcome, it appears that planting in landscape-scale restoration projects need not be constrained by seasonal factors.

Longitude, latitude and slope were identified as factors influencing restoration success in some models. Low restoration success at lower latitude sites, most of which are associated with the inland fringes of spekboom thicket, can be ascribed to a higher frost exposure there than at lower-latitude sites closer to the coast (Duker et al., 2015a; Duker et al., 2020). There was a trend for sites in the eastern (>25°E) part of the study domain to perform better than those in the west, suggesting a positive influence of higher warm-season precipitation. Based on these relationships, it is reasonable to generalise that the odds of good restoration outcomes will decrease from the southeast (coastal areas with the highest proportion of warm-season precipitation) to the northwest (inland areas with the highest proportion of cool-season precipitation).

It goes without saying that differences in project management can massively influence the outcome of any enterprise, including restoration projects (*e.g.* McConnachie et al., 2012). However, we retrieved ambiguous results with regards to managers, with each of the three teams performing better or worse in different contexts.

### Variables not under management control

Factors beyond the control of managers, for example droughts and cold snaps, can significantly affect the efficacy of restoration projects (Harris et al., 2006; Halme et al., 2013). We found that including these in models had a greater impact on survivorship than carbon sequestration, albeit with some surprising results. Three rule sets, for example, showed that receiving more than ca. 233 mm in the first year after planting—an amount that approximates the average rainfall of much of the spekboom thicket region (Vlok, Euston-Brown & Cowling, 2003)—had a negative effect on survivorship. However, rainfall amount in the first month after planting was positively correlated with carbon sequestration. Given that the analysis of different spekboom planting regimes implemented in the plots showed that applying water at the time of planting did not improve survivorship or carbon sequestration (Van der Vyver, Mills & Cowling, 2021), it is likely that watering of truncheons a few weeks after planting, when they are rooting, could have a positive effect on survivorship and growth. More research is required on this topic.

Direct evidence of frost damage, as well as indirect indicators such as position in landscape and slope (Duker et al., 2020), emerged as important predictors of restoration success in all the models. This is not surprising given the vulnerability of spekboom to frost damage (Duker et al., 2015a). We suspect that frost damage is mainly linked to the location of plots outside of target habitat (Duker et al., 2020), reinforcing the importance of the careful identification of target habitat for restoration.

No clear patterns emerged regarding the impact of soil factors on the success of spekboom restoration. Soil Mg (cation exchange capacity) above a value of ca 3.2 cmolc kg^−^^1^ had a positive effect on carbon sequestration, as did lower soil K and silt content. Some of these edaphic features may reflect impacts of past degradation (Lechmere-Oertel, Kerley & Cowling, 2005; Rutherford, Powrie & Husted, 2014) and could suggest that some degraded sites have exceeded edaphic thresholds for effective restoration. This topic requires more research.

### Lesson learnt for project management

An important lesson to be learnt from our study is that scientists need to collaborate with managers and implementors throughout the duration of a project; without this, learning is greatly constrained (Cowling et al., 2008; Halme et al., 2013; Mills et al., 2015). The committee that oversaw the project should have insisted on the appointment of an appropriately skilled scientist to ensure effective implementation in the field. This may have prevented the large-scale misidentification of target habitat and corrected for problems associated with data collection, database management, herbivore exclusion and experimental design. The project ran for almost five years before any direct scientific oversight was imposed. This was a grave mistake.

Finally, more effort should have been made by managers to assess the willingness of landowners to participate in the experiments and therefore, develop a sense of ownership of the project (cf. Knight et al., 2010). Excluding landowners unwilling to collaborate may have ensured better overall plot monitoring and maintenance, and therefore, a lower browsing impact.

## Conclusions

Our study revealed several important insights for improving the success of spekboom restoration at the regional scale. These are:

• Potential restoration sites must be located in target habitat identified in the field by experts or guided by accurate maps at the 1:10,000 scale or less. Producing these maps will be difficult and time-consuming. There is likely no substitute for an expert plant ecologist in the delineation of suitable habitat for the restoration of spekboom thicket.
• Many of the topographic factors that emerged as influential in the models (*e.g.* eschew bottomlands in favour of sloping ground) are ultimately surrogates for frost exposure, and hence non-target habitat.
• Topographic and management factors predicted carbon sequestration better than survivorship, which was more strongly influenced by rainfall and soil variables.
• Small restoration sites must be protected from herbivory, especially browsing by indigenous antelope. This may prove costly if game-proof fencing turns out to be the only solution to this problem.
• Fortuitously, only a handful of factors beyond the realm of management emerged as having a positive effect on restoration success. These included higher rainfall in the first month after restoration, which could be simulated by watering planted truncheons during this time. Soil variables were largely associated with ambiguous outcomes.
• Planting on equator-facing slopes during the warmer months should be avoided.

##  Supplemental Information

10.7717/peerj.11944/supp-1 Supplemental Information 1 Explanation of the acronyms for all explanatory variables used in the study

10.7717/peerj.11944/supp-2 Supplemental Information 2 Photographs of truncheons exposed to frost and to browsing, illustrating signs of the cause of impact on observed truncheons Photographs of truncheons exposed to frost (a, b, c) and to browsing (d, e, f), illustrating signs of the cause of impact on observed truncheons.

10.7717/peerj.11944/supp-3 Supplemental Information 3 Photographs of example thicket-wide plots exposed to four different browsing intensity categories used as the browse variable in our models Examples of plots identified as experiencing high (4) browse intensity are shown in subfigures a, b, c; Medium intensity plots by subfigures d, e, f; Low intensity browsing by g, h, i and j. Very low intensity browsing is shown by photographs of plots shown in subfigures k and l.

10.7717/peerj.11944/supp-4 Supplemental Information 4 Raw data and R code for deriving results, tables and graphs presented in the manuscript The files df173.RData and df83.RData refer to the two separate datasets analyzed in the article and analyzed by the accompanying code file (code.R) to provide the results, tables (Table 1) and graphs (Figs. 5–10) presented in the article.

10.7717/peerj.11944/supp-5 Supplemental Information 5 Analyses of extra variables added to our original models

## Additional Information and Declarations

### Field Study Permissions

The following information was supplied relating to field study approvals (i.e., approving body and any reference numbers): No Field Permit system was in place at the time of sampling. Our experimental plots have been established exclusively on private land with the mutual understanding that they will be monitored from time to time by appointed managers and service providers to the project. These agreements with the landowners have been made prior to the establishment (circa pre-2008) of the plots and mostly on a verbal basis. Verbal permission from landowners or authorized family members of landowners and/or managers were given to sampling teams on an *ad hoc* basis. A relatively current list of landowners for each of the plots used within our study is available in the Supplemental files.

### Data Availability

The following information was supplied regarding data availability: The raw data for each of the two datasets analyzed are available as Supplemental Files.
