# Supplementary material for: Herbivory and misidentification of target habitat constrain region-wide restoration success of spekboom (Portulacaria afra) in South African subtropical succulent thicket
Source: PeerJ. 2021 Aug 11;9:e11944. doi: 10.7717/peerj.11944 (PMC8364318; doi:10.7717/peerj.11944)
Supplement: Supplemental Information 1 [file peerj-09-11944-s001.doc]

| **abbv.** | **description** | **type** |
| --- | --- | --- |
| age | number of months since month of planting | numerical |
| aspect | Recorded with a compass as one of 16 compass points in the middle of plot | categorical |
| slope | Slope steepness estimated in 5° intervals on a 10-point scale (0 - 45°) | numerical |
| landf | Landform in 4 categories : 1=Bottomlands, 2=Footslope, 3=Midslope, 4=Topslope or Crest | numerical |
| elev | Elevation in metres | numerical |
| lon | Longitude in decimal degrees, representing the increase in summer rainfall towards the East | numerical |
| lat | Latitude in decimal degrees, inidicative of the moisture gradient associated with distance from the coast to the South | numerical |
| STEPvt | Spekboom-rich vegetation as identified by the STEP conservation plan (Vlok et al. 2003). *Spek* refers to areas mapped as spekboom thicket, *Mosaic* and *NoSpek* are areas where spekboom thicket is not a dominant component. | categorical |
| habitat | Habitat for spekboom-rich vegetation identified on site. *Spek* refers to degraded spekboom thicket, *EtSpek* refers to the ecotone between spekboom thicket and adjacent vegetation and *noSpek* refers to vegetation where naturally spekboom is not a dominant component | categorical |
| browse | Browse intensity on a numeric 4-tiered scale where 1=Imperceptible, 2=Light, 3=Moderate and 4=Heavy | integer |
| wlife | Are there signs of wildlife present within plot ? | binary |
| domE | Are there evidence of domestic animals entering the exclosure ? | binary |
| seasonP | Season within which the plot was planted (four categories) | categorical |
| frostb | Any signs of frostburn on spekboom truncheons in plot? | binary |
| holefence | Are there any holes evident in the fence enclosure large enough for herbivores to access? | binary |
| gateO | Was the entrance gate to the exclosure left open when visited? | binary |
| fenceC | The condition or integrity of the exclosure fence measured on a numerical scale (1=fence completely removed, 5=As the day it was erected or better) | numerical |
| manager | Project manager under which plot was planted (*Y*, *P* or *S*) | categorical |
| mm1mnt | Total rainfall during the month within which the plot was planted | numerical |
| mm3|6|12mnt | Cumulative rainfall after 3|6|12 months since plot was planted | numerical |
| summ6|12|18 | Cumulative rainfall during the summer months (Dec-Feb) after 6|12|18 months since plot was planted | numerical |
| atm6|12|18 | Cumulative rainfall during the autumn months (Mar-May) after 6|12|18 months since plot was planted | numerical |
| wnt6|12|18 | Cumulative rainfall over the winter months (Jun-Aug) after 6|12|18 months since planting | numerical |
| spr6|12|18 | Cumulative rainfall over the spring months (Sep-Nov) after 6|12|18 months since planting | numerical |
| sumr6|12|18 | Cumulative rainfall over the warmer months (Sep-Feb) after 6|12|18 months since planting | numerical |
| wint6|12|18 | Cumulative rainfall over the coooler months (Mar-Aug) after 6|12|18 months since planting | numerical |
| frostb | Are there any indications of frost burn on planted cuttings ? | binary |
| pH | Soil pH, using the potassium chloride method | numerical |
| stone | Stone volume percentage, using water displacement | numerical |
| P_mg | Soil P using the Bray II method, but if pH > 7.0 the Olsen method was used (mg.kg^−1^) | numerical |
| K_mg | Extractable K (Colwell) in mg.kg^−1^ | numerical |
| Na_mg | Na cation exchange capacity (cmolc.kg^−1^) | numerical |
| K1_mg | K cation exchange capacity (cmolc.kg^−1^) | numerical |
| Ca_mg | Ca cation exchange capacity (cmolc.kg^−1^) | numerical |
| Mg_mg | Mg cation exchange capacity (cmolc.kg^−1^) | numerical |
| C_pc | % Carbon, using Walkley-Black method | numerical |
| Na_pc | % base Na saturation | numerical |
| K_pc | % base K saturation | numerical |
| Ca_pc | % base Ca saturation | numerical |
| Mg_pc | % base Mg saturation | numerical |
| N_pc | % soil Nitrogen | numerical |
| clay | % clay from soil texture analysis (mechanical method) | numerical |
| silt | % silt from soil texture analysis (mechanical method) | numerical |
| sand | % sand from soil texture analysis (mechanical method) | numerical |
