# Supplementary material for: Herbivory and misidentification of target habitat constrain region-wide restoration success of spekboom (Portulacaria afra) in South African subtropical succulent thicket
Source: PeerJ. 2021 Aug 11;9:e11944. doi: 10.7717/peerj.11944 (PMC8364318; doi:10.7717/peerj.11944)
Supplement: Supplemental Information 2 — Photographs of truncheons exposed to frost (a, b, c) and to browsing (d, e, f), illustrating signs of the cause of impact on observed truncheons. [file peerj-09-11944-s002.pdf]

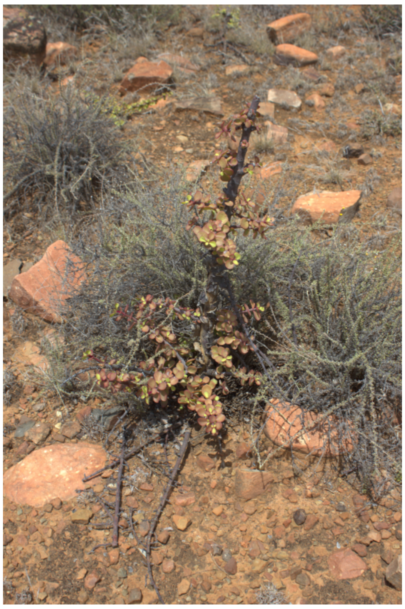

(a) Frost exposure: red discoloration of leaves

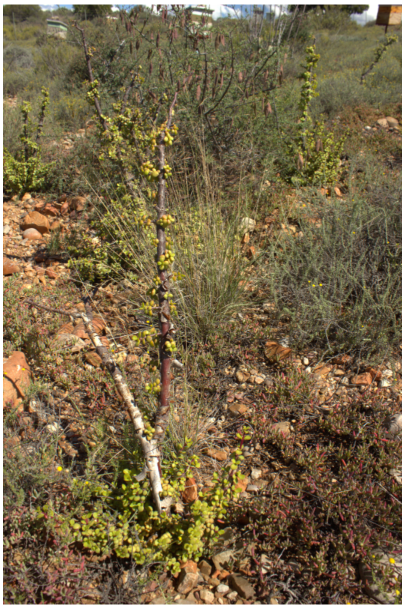

(b) Frost exposure: cracked and flaked stem with small leaves

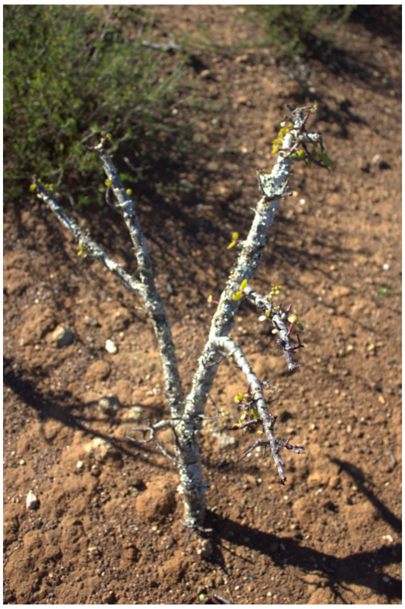

(c) Frost exposure: cracked and lichen-covered stem with few leaves

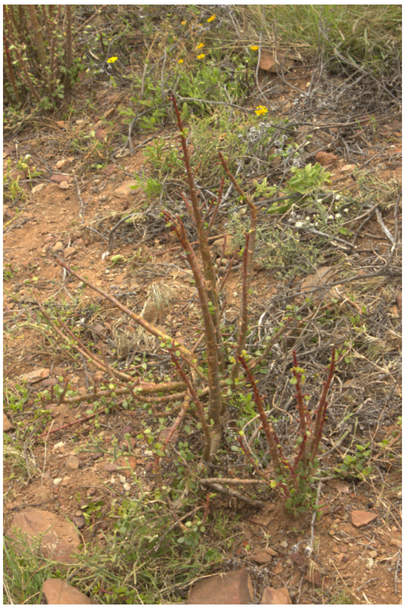

(d) Browse exposure: leaves stripped off healthy stems

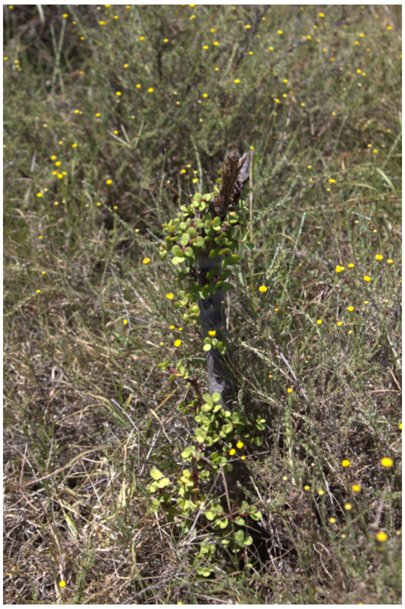

(e) Browsing induced damage. Notice healthy leaves still emerging

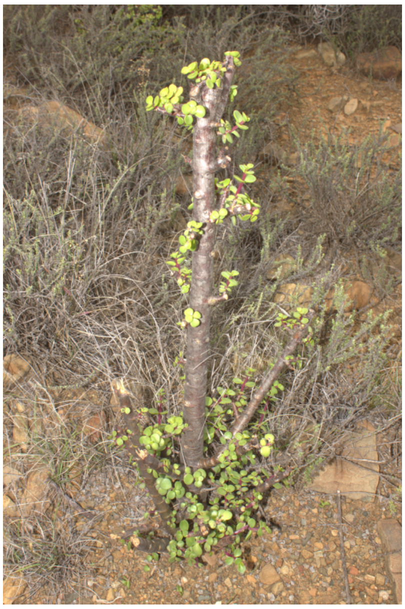

(f) Browsing induced damage: top of main stem and side shoots missing

Figure S1: Photographs of truncheons exposed to frost (a, b, c) and to browsing (d, e, f) illustrating signs of the cause of impact on observed truncheons.
