# Supplementary material for: Herbivory and misidentification of target habitat constrain region-wide restoration success of spekboom (Portulacaria afra) in South African subtropical succulent thicket
Source: PeerJ. 2021 Aug 11;9:e11944. doi: 10.7717/peerj.11944 (PMC8364318; doi:10.7717/peerj.11944)
Supplement: Supplemental Information 3 — Examples of plots identified as experiencing high (4) browse intensity are shown in subfigures a, b, c; Medium intensity plots by subfigures d, e, f; Low intensity browsing by g, h, i and j. Very low intensity browsing is shown by photographs of plots shown in subfigures k and l. [file peerj-09-11944-s003.pdf]

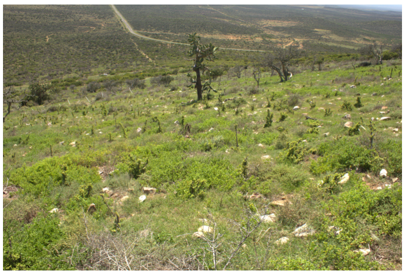

(a) High (4) browse intensity

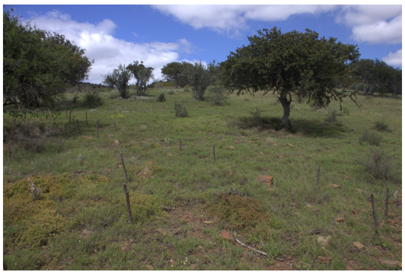

(b) High (4) browse intensity

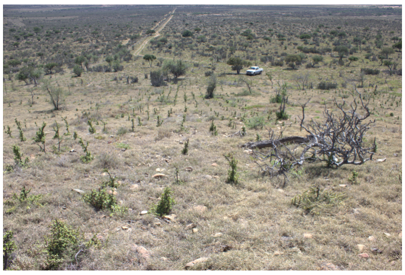

(c) High (4) browse intensity

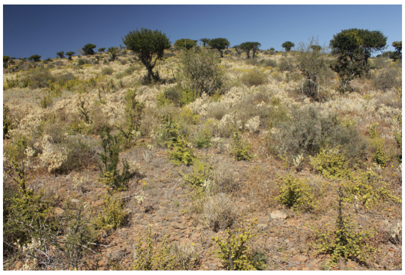

(d) Medium (3) browse intensity

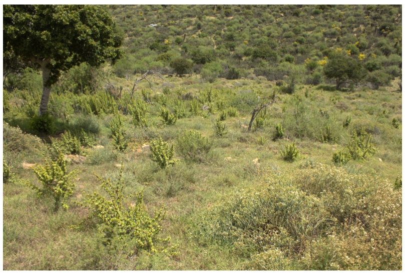

(e) Medium (3) browse intensity

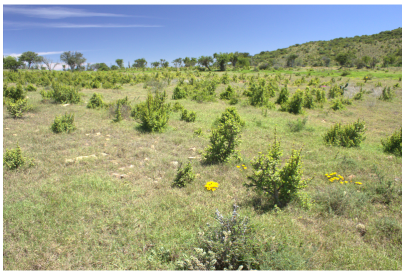

(f) Medium (3) browse Intensity

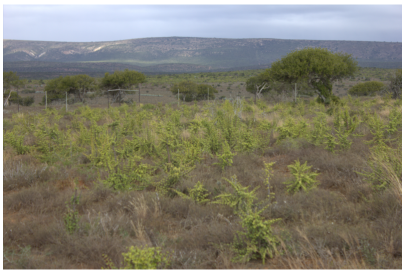

(g) Low (2) browse intensity

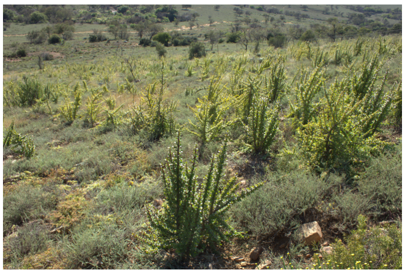

(h) Low (2) browse intensity

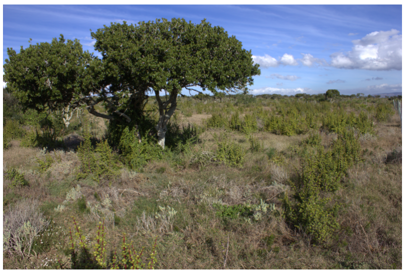

(i) Low (2) browse intensity

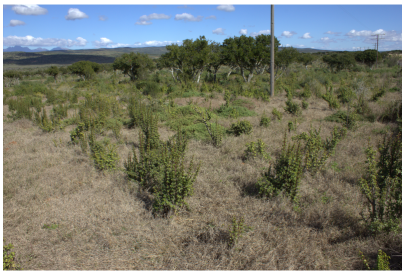

(j) Low (2) browse intensity

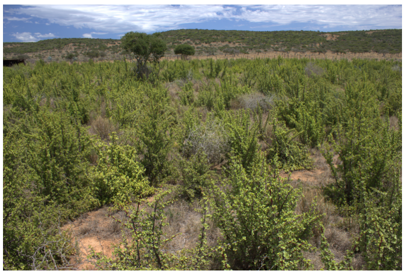

(k) Very low (1) browse intensity

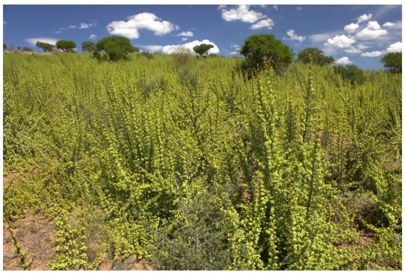

(l) Very low (1) browse intensity

Figure S2: Photographs of thicket-wide plots attributed to four different browsing intensity categories used in our models. Examples of plots identified as experiencing high (4) browse intensity are shown in subfigures a, b, c; Medium intensity plots by subfigures d, e, f; Low intensity browsing by g, h, i and j. Very low intensity browsing is shown by photographs of plots shown in subfigures k and l.
