# Supplementary material for: Herbivory and misidentification of target habitat constrain region-wide restoration success of spekboom (Portulacaria afra) in South African subtropical succulent thicket
Source: PeerJ. 2021 Aug 11;9:e11944. doi: 10.7717/peerj.11944 (PMC8364318; doi:10.7717/peerj.11944)
Supplement: Supplemental Information 5 [file peerj-09-11944-s005.pdf]

Herbivory and misidentification of target habitat constrain region-wide restoration success of spekboom (Portulacaria afra) in South African subtropical succulent thicket

- Additional Analyses with extra topographic and rainfall variables -

Marius van der Vyver

April 5, 2021

Contents

1 **Introduction** 1

2 **Method** 2

3 **Results** 2

4 **Discussion** 9

5 **Conclusion** 9

1 Introduction

Both reviewers questioned the absence of a number of variables in the models presented in our original manuscript. Reviewer 2 suggested the use of a range of topographic variables derived from digital elevation models, while Reviewer 1 questioned why rainfall variables of 1 and 3 months preceding the planting were not used in the original models. Here we show the results of a rerun of our models with these suggested variables included.

The list of variables added (see Table 1) were added to the original variables (see Table 1 in the submitted manuscript) in this additional analysis.

Table 1: A descriptive table of the extra predictor variables fitted along with those outlined in Table 1.

**abbv.**	**description**	**type**
TPI	Topographic Position Index - is the difference between the value of a cell and the mean value of its 8 surrounding cells (*5*).	numerical
flowdir	returns the 'flow direction' (of water), i.e. the direction of the greatest drop in elevation (or the smallest rise if all neighbors are higher) (*1*). Encoded as powers of 2 (0 to 7).	numerical
TRI	Terrain Ruggedness Index - is the mean of the absolute differences between the value of a cell and the value of its 8 surrounding cells (*5*).	numerical
roughness	the difference between the maximum and the minimum value of a cell and its 8 surrounding cells (*5*).	numerical
hillS	hill shade from slope and aspect layers (both in radians) (*2*).	numerical
aspect2	Calculated from ASTER DEM with the Horn (*2*) algorithm, considered best for rough surfaces (radians)	numerical
slope2	Calculated from ASTER DEM with the Horn (*2*) algorithm, considered best for rough surfaces (radians).	numerical
planform	The planform curvature is the second derivative(s) of the elevation surface (slope of the slope) and perpendicular to the direction of the maximum slope (*6*).	numerical
McNab	McNab's variant of the surface curvature (concavity/convexity) index (*3*, *4*).	numerical
mmtpre1|3	Cumulative rainfall 1|3 months before plot was planted, including month planted	numerical

2 Method

The new models presented here (see Table 1, original model names appended with "b"), replicated from those presented in our manuscript. We added the newly generated variables outlined in Table 1 to those outlined in Table S1 in the original manuscript. To our original models originally fitted with variables related to topographic and management factors, an additional nine topographic variables were added for both survival and carbon sequestration as response variables.

To the orignal models fitted with all available variables, these newly derived topographic variables were also added, together with the two additional rainfall variables (i.e. total rainfall one and three months before planting respectively).

Table 1 provides a description and outline of each of these new variables. Figures 2-5 illustrates the nine most important rule sets for each of the models with the highest percentage variance explained (highlighted in Table 2.)

3 Results

A comparison of the variance explained between the original and new models with extra variables (Table 1 in the original manuscript vs Table 2 here) reveal only a marginal increase of the survival models and no change regarding the carbon sequestration models as a result of the added variables. Such marginal increases are usually expected when adding more variables to a predictive model, irrespective of how well they are able to predict a specific response. This suggest that the newly added variables are not better predictors than the ones used in our original manuscript.

Similar sets of variables were identified as important predictors of both survival and carbon sequestration (See Figure 1) and both browse intensity and habitat again emerged as highly influential. The latter more in terms of the carbon sequestration models than the survival models. Of the newly added variables, it was flow direction which emerged as an important predictor of survival, while the planform curvature and amount of rain recorded a month prior to planting also proved to be of some importance as predictors of carbon sequestration. But these were found not as important as those variables already identified in original models.

The decision trees depicted in Figures 2-5 do not produce more nuanced splits as suggested by reviewer 2, but show similar rules idenitified from the first models in the original manuscript. Flow direction, planform curvature and TPI (topographic position index) were new important rule components identified by the new models. These digitally derived variables however could not trump our ground-truthed variables for model fitting - as used in our original models.

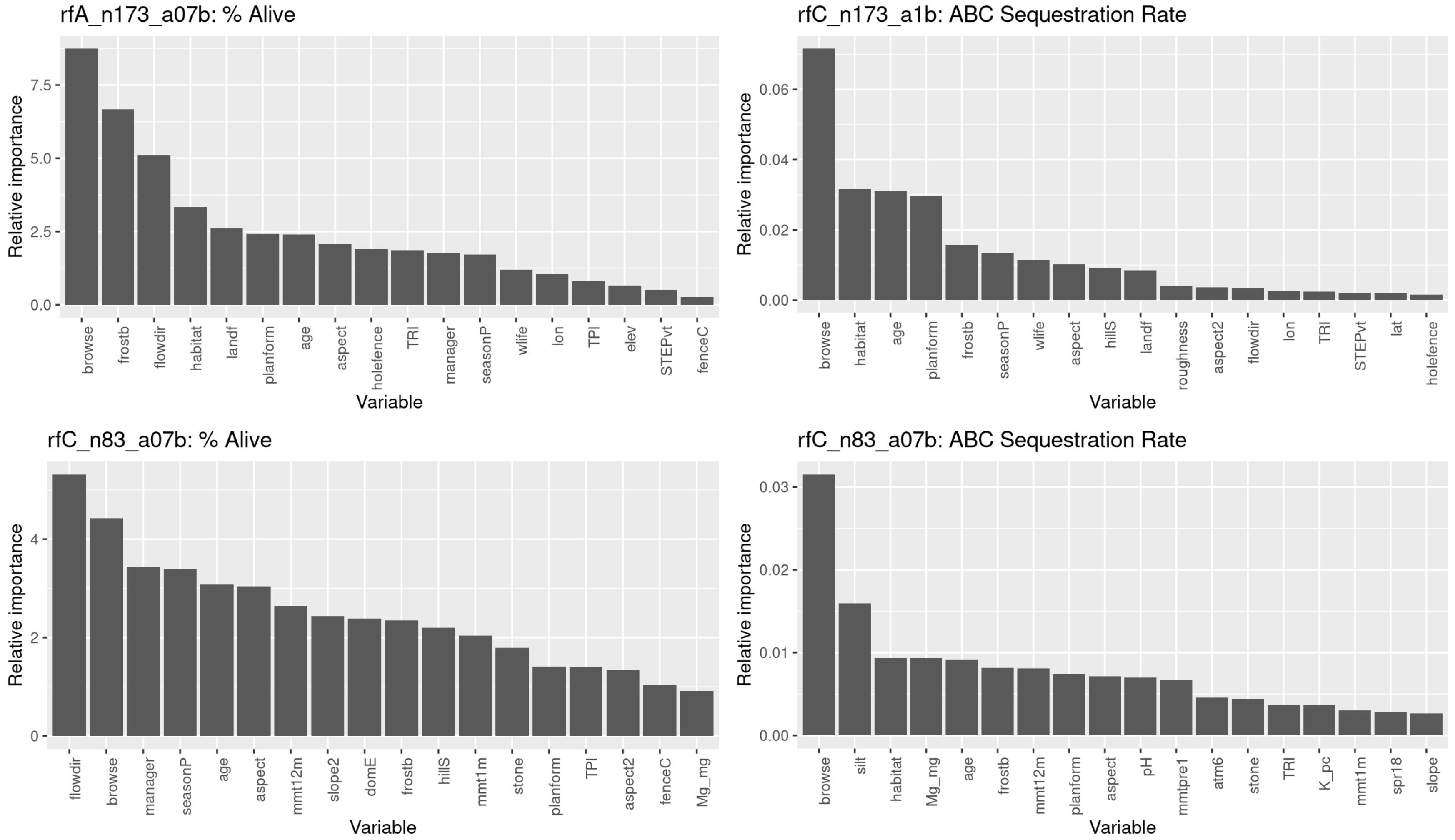

Figure 1: Variable importance plots for the four models with the highest variances explained (see Table 2).

Table 2: Model statistics for each of the models developed with additional variables outlined in Table 1.

model	pen	resp	nplot	nvar	n	MAE	RMSE	RMSE.n	sd.n	%VarExpl	cv.MSE	cv.MSE.se	cv.MAE	cv.MAE.se
rfA_n173_a0b	0.00	pcAlive	173	27	535	11.25	13.80	0.68	0.62	0.54	2.66e+02	1.57e+01	1.30e+01	4.27e-01
rfA_n173_a07b	0.70	pcAlive	173	27	535	11.05	13.73	0.68	0.63	0.54	2.49e+02	1.45e+01	1.27e+01	4.03e-01
rfA_n173_a1b	1.00	pcAlive	173	27	535	9.11	11.42	0.56	0.76	0.68	2.70e+02	1.71e+01	1.31e+01	4.30e-01
rfA_n83_a0b	0.00	pcAlive	83	68	254	9.77	12.57	0.63	0.62	0.6	2.67e+02	2.50e+01	1.29e+01	6.30e-01
rfA_n83_a07b	0.70	pcAlive	83	68	254	8.79	11.36	0.57	0.72	0.68	2.38e+02	2.36e+01	1.21e+01	6.02e-01
rfA_n83_a1b	1.00	pcAlive	83	68	254	9.28	11.78	0.59	0.69	0.65	2.25e+02	2.26e+01	1.17e+01	5.92e-01
rfC_n173_a0b	0.00	ABCsrt	173	27	535	0.01	0.02	0.57	0.70	0.67	7.29e-04	1.82e-04	1.52e-02	9.66e-04
rfC_n173_a07b	0.70	ABCsrt	173	27	535	0.01	0.02	0.44	0.84	0.8	6.89e-04	1.59e-04	1.53e-02	9.22e-04
rfC_n173_a1b	1.00	ABCsrt	173	27	535	0.01	0.01	0.40	0.88	0.84	4.64e-04	7.94e-05	1.40e-02	7.10e-04
rfC_n83_a0b	0.00	ABCsrt	83	67	254	0.02	0.04	0.84	0.26	0.3	1.61e-03	5.55e-04	2.14e-02	2.13e-03
rfC_n83_a07b	0.70	ABCsrt	83	68	254	0.01	0.01	0.34	0.88	0.88	1.07e-03	4.08e-04	1.65e-02	1.78e-03
rfC_n83_a1b	1.00	ABCsrt	83	68	254	0.01	0.02	0.35	0.88	0.88	4.98e-04	1.06e-04	1.41e-02	1.09e-03

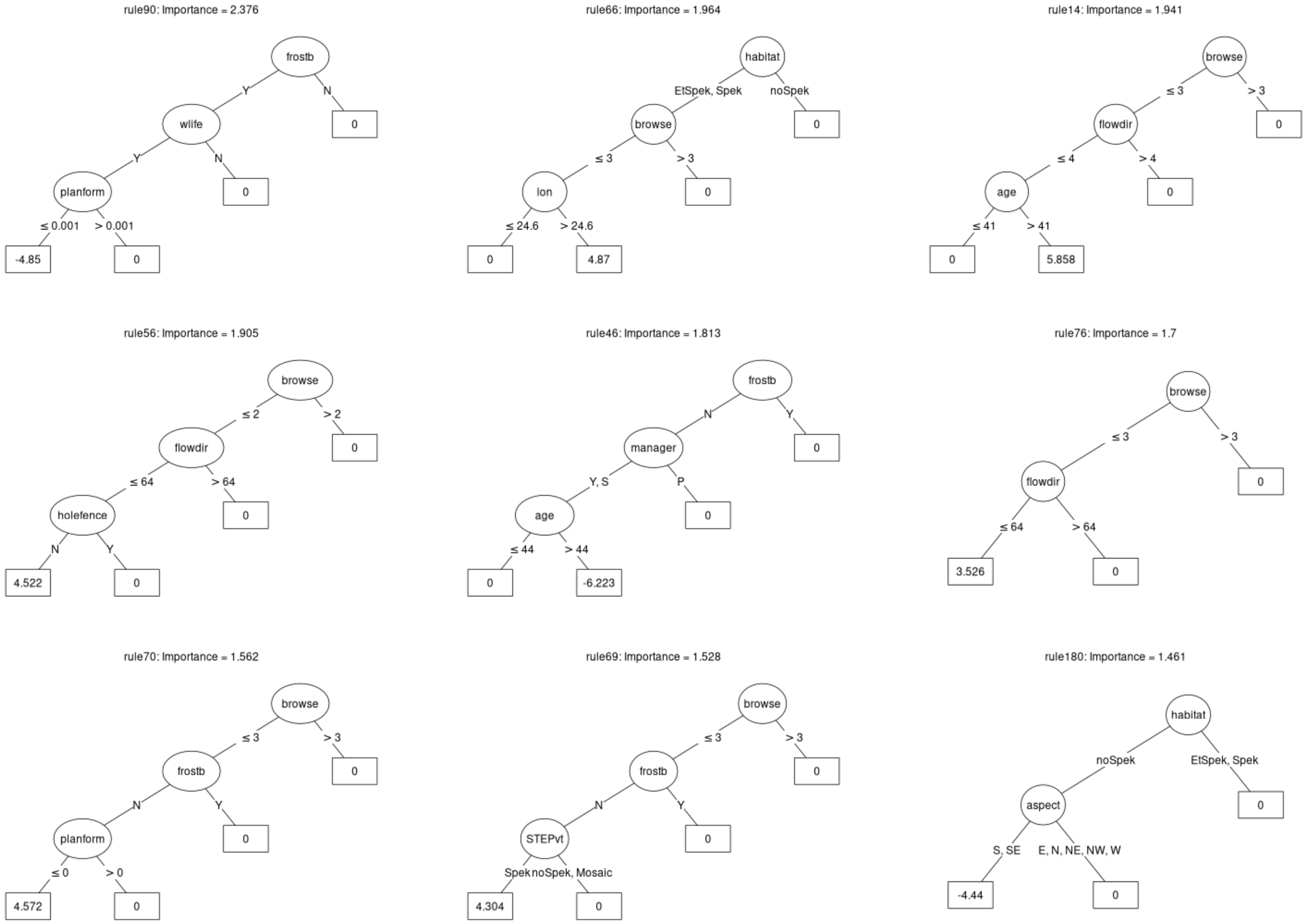

Figure 2: The nine most important rules identified by model rfA-n173-a07b (see Table 2).

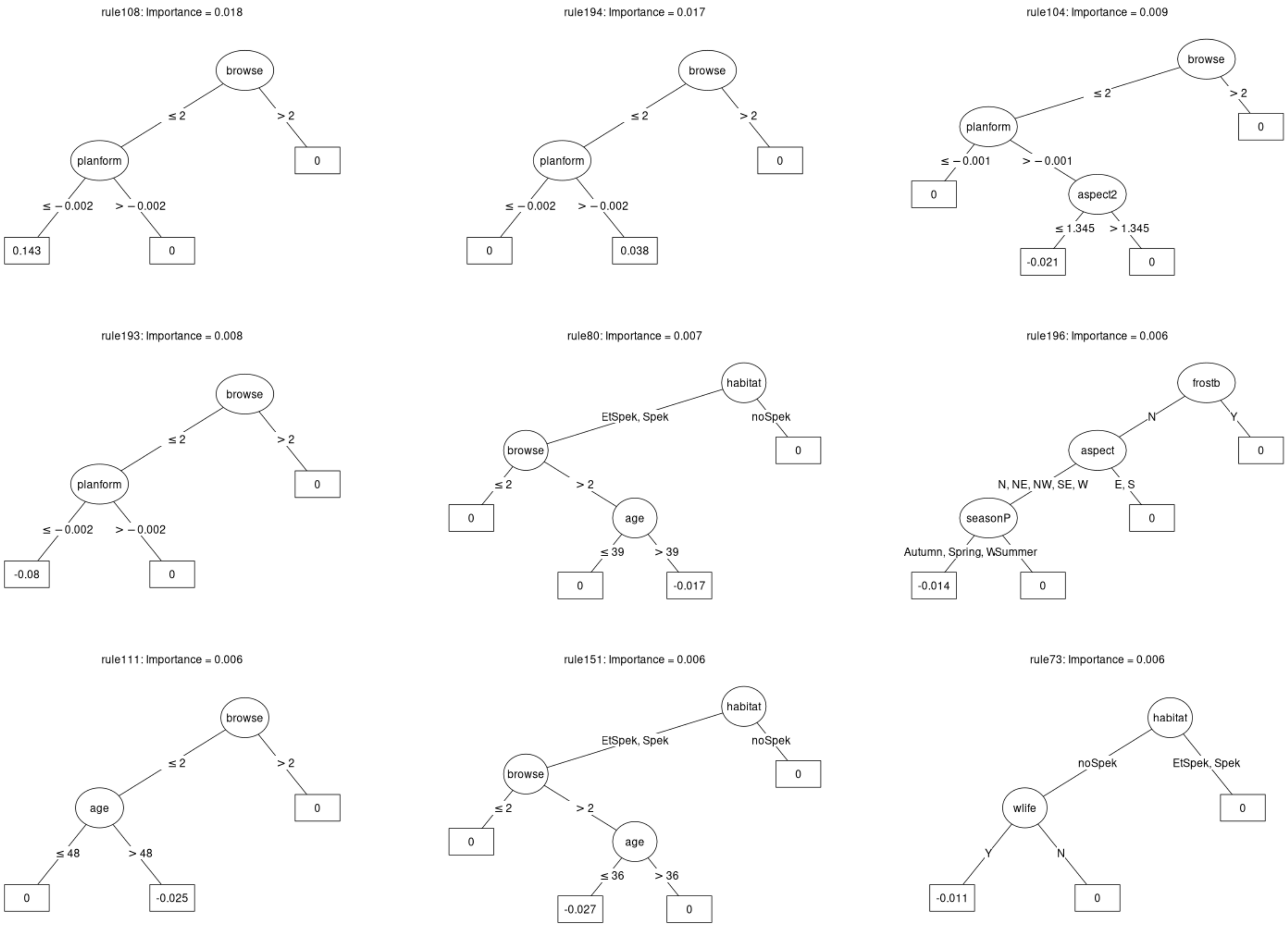

Figure 3: The nine most important rules identified by model rfC-n173-a1b (see Table 2).

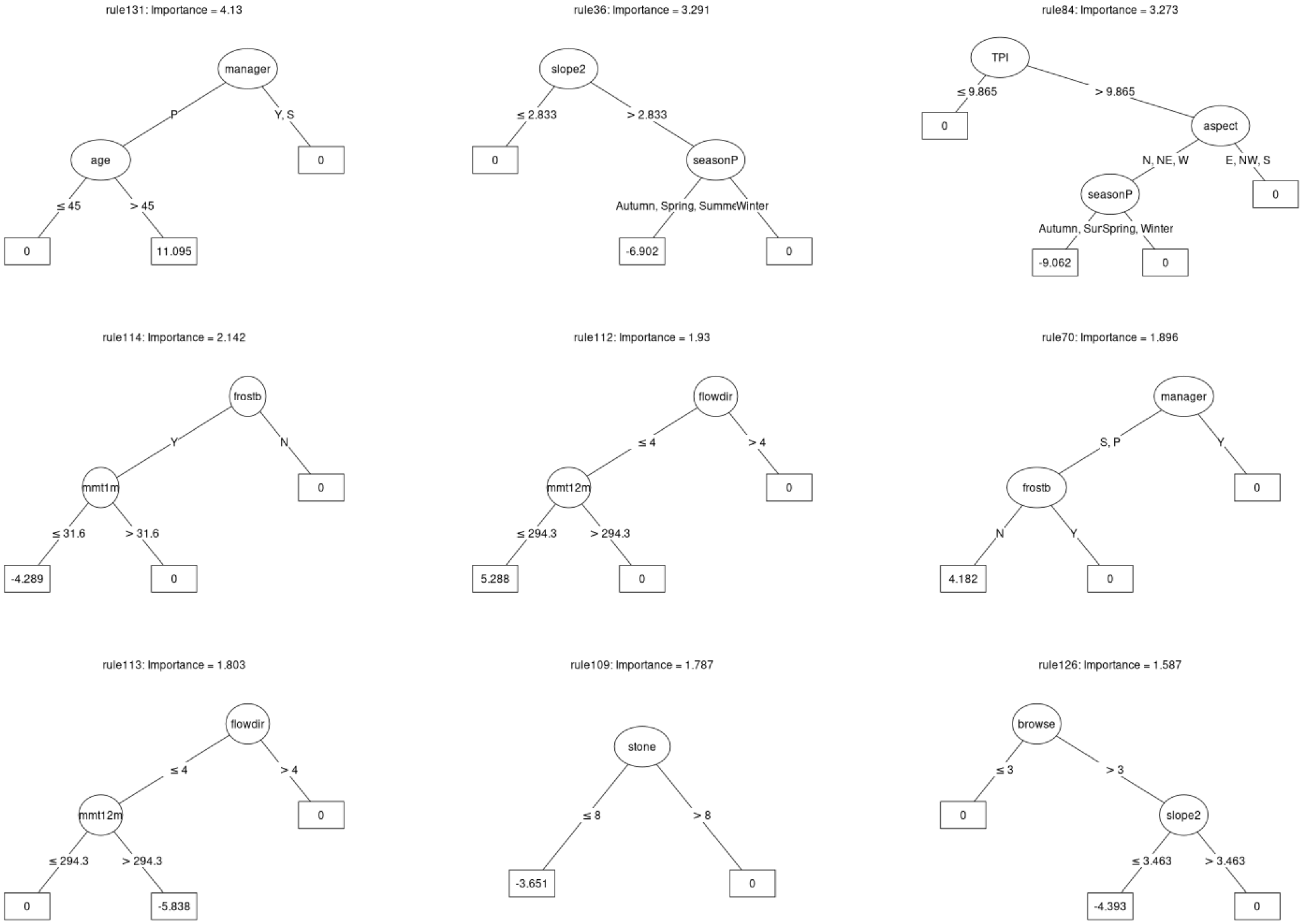

Figure 4: The nine most important rules identified by model rfA-n83-a07b (see Table 2).

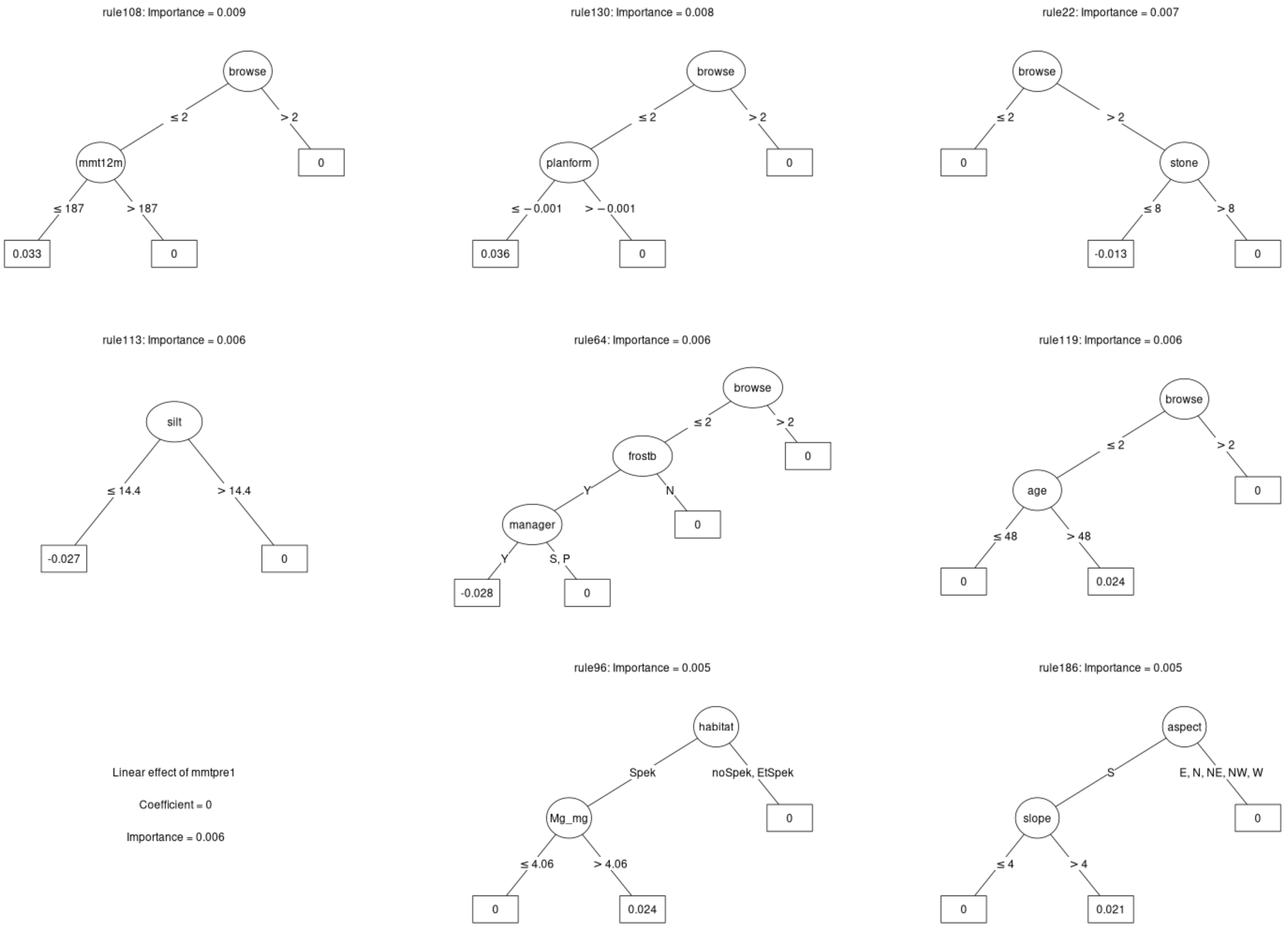

Figure 5: The nine most important rules identified by model rfC-n83-a07b (see Table 2).

4 Discussion

Since there is little improvement in the variance explained between the models fitted in the original manuscript and those presented here with the added suggested predictors, the new models are not more accurate than those originally fitted.

The derived topographic variables added as suggested by Reviewer 1 did not significantly change the accuracy of the models, although it may have identified new variables that could be used interchangeably with some those selected originally. Two of the new variables (flow direction and planform curvature) could be useful for future potential mapping of target habitat before planting, but more research is needed in this regard. These variables are certainly useful in technical habitat modeling, but requires digital processing from satelite derived data. Since the aim of our paper was to identify variables easily identified in the field by restoration practitioners, we did not include the new variables in our models as they do not provide better results than our field-derived variables.

5 Conclusion

The fitting of additonal new derived variables as suggested by the reviewers did not produce better models or better predictor variables for either survival or carbon sequestration than those identified in the original models and manuscript.

References

1. R. J. Hijmans, *raster: Geographic Data Analysis and Modeling*, R package version 3.4-5.
2. B. K. Horn, *Proceedings of the IEEE* **69**, 14–47 (1981).
3. W. H. McNab, *Forest Science* **35**, 91–104 (1989).
4. W. H. McNab, *Canadian Journal of Forest Research* **23**, 1100–1107 (1993).
5. M. F. Wilson, B. O'Connell, C. Brown, J. C. Guinan, A. J. Grehan, *Marine Geodesy* **30**, 3–35 (2007).
6. L. W. Zevenbergen, C. R. Thorne, *Earth surface processes and landforms* **12**, 47–56 (1987).
